# Systematics of the parasitic wasp genus *Oxyscelio* Kieffer (Hymenoptera, Platygastridae s.l.), part III: African fauna

**DOI:** 10.3897/zookeys.565.7185

**Published:** 2016-02-17

**Authors:** Roger A. Burks, Lubomír Masner, Norman F. Johnson, Andrew D. Austin

**Affiliations:** 1 Department of Evolution, Ecology, and Organismal Biology, The Ohio State University, 1315 Kinnear Road, Columbus, OH 43212, U.S.A.; 2 Agriculture and Agri-Food Canada, K.W. Neatby Building, Ottawa, ON K1A 0C6, Canada; 3 Australian Centre for Evolutionary Biology and Biodiversity, School of Biological Sciences, The University of Adelaide, SA 5005, Australia

**Keywords:** Platygastroidea, Scelionidae, Oxyscelio, Scelioninae, key, revision, database, parasitoid

## Abstract

African species of *Oxyscelio* (Hymenoptera: Platygastridae s.l.) are revised. A total of 14 species are recognized, 13 of which are described as new: *Oxyscelio absentiae* Burks, **sp. n.**, *Oxyscelio galeri* Burks, **sp. n.**, *Oxyscelio gyri* Burks, **sp. n.**, *Oxyscelio idoli* Burks, **sp. n.**, *Oxyscelio intensionis* Burks, **sp. n.**, *Oxyscelio io* Burks, **sp. n.**, *Oxyscelio kylix* Burks, **sp. n.**, *Oxyscelio lunae* Burks, **sp. n.**, *Oxyscelio nemesis* Burks, **sp. n.**, *Oxyscelio pulveris* Burks, **sp. n.**, *Oxyscelio quassus* Burks, **sp. n.**, *Oxyscelio teli* Burks, **sp. n.** and *Oxyscelio xenii* Burks, **sp. n.** The genus *Freniger* Szabó, **syn. n.** is recognized as part of an endemic African species group of *Oxyscelio* with incomplete hind wing venation, and *Oxyscelio bicolor* (Szabó), **comb. n.** is therefore recognized as the only previously described species of *Oxyscelio* from Africa. The *Oxyscelio crateris* and *Oxyscelio cuculli* species groups, previously known from southeast Asia, are represented in Africa by seven and one species respectively.

## Introduction

*Oxyscelio* Kieffer was first described to contain a single species of Scelioninae from Java (Kieffer 1907). It remained in obscurity until Dodd (1931) recognized that it was the oldest generic name corresponding to a set of Australian and Indo-Malayan species that previously had been placed in several other genera. Dodd’s concept of *Oxyscelio* has been upheld in more recent examinations of scelionine genera (Masner 1976, Galloway and Austin 1984, Austin and Field 1997). The Indo-Malayan, Palearctic (Burks et al. 2013a, Johnson et al. 2013), Australian, and Pacific (Burks et al. 2013b) species of *Oxyscelio* have been recently reviewed, expanding the number of described species of the genus from 36 to 170, while retaining as valid all but one of the species that had been recognized by Dodd.

Despite the diversity of *Oxyscelio*, very little is known of its life history. The host of *Oxyscelio perpensus* Kononova, an exposed orthopteran egg laid from an unknown species onto plant tissue, was photographed as part of its original description (Kononova and Fursov 2007) and is the only known host record of the genus.

In this study we recognize 14 species of *Oxyscelio* from the Afrotropical realm, including 13 newly described species. Eight of these species are placed in species groups previously recognized from the Indo-Malayan realm. Four of the remaining species are placed in a uniquely African species group comprising the only species of *Oxyscelio* known to have incomplete hind wing venation, a feature that has most notably been found in other genera of Scelioninae, including *Scelio* Latreille, *Sparasion* Latreille, and *Nixonia* Masner. These species are determined to belong to *Oxyscelio* based on a single spur on both the mid and hind tibia, the presence of a facial submedian carina, and fore wing with a punctiform marginal vein and no pseudostigma (*sensu* Masner 1976).

## Materials and methods

Specimens examined were provided by the following collections: Australian National Insect Collection, Canberra, Australia (ANIC)^1^; The Natural History Museum, London, United Kingdom (BMNH)^2^; Canadian National Collection of Insects, Arachnids and Nematodes, Ottawa, Canada (CNCI)^3^; Hungarian Natural History Museum, Budapest, Hungary (HNHM)^4^; Museum of Comparative Zoology, Harvard University, Cambridge, Massachusetts, USA (MCZC)^5^; Lund Museum of Zoology, Lund University, Lund, Sweden (MZLU)^6^; National Museum of Kenya (NMKE)^7^; C.A. Triplehorn Insect Collection, Ohio State University, Columbus, Ohio (OSUC)^8^; Queensland Primary Industries and Fisheries Insect Collection, Indooroopilly, Australia (QDPC)^9^; Queensland Museum, Brisbane, Australia (QMBA)^10^; South African Museum, Iziko Museums of Cape Town, South Africa (SAMC)^11^; National Museum of Natural History, Washington, DC (USNM)^12^; Waite Insect and Nematode Collection, Adelaide, Australia (WINC) ^13^.

This revision is a product of the Platygastroidea Planetary Biodiversity Inventory, funded by the U.S. National Science Foundation (N.F. Johnson, Ohio State University; Andy Austin, University of Adelaide; Principal Investigators). An objective of this project is to use biodiversity informatics resources to accelerate taxonomic work, making real-time collaboration possible. Data associated with specimens examined in this study can be accessed at hol.osu.edu and entering the unique specimen identifier (e.g. OSUC 359541) in the search form. Scale bars on all figures are in mm format. Morphological terminology follows Mikó et al. (2007) except as specified here. Ovipositor terminology is used as described by Austin and Field (1997). “T1 midlobe” refers to the raised antero-medial area of T1 that is flanked by depressed lateral areas. This is usually flat and only weakly elevated in *Oxyscelio*, and therefore is not strictly the same as a T1 horn, but a T1 midlobe can be expressed as a T1 horn. All terms except those for surface sculpture are defined in the Hymenoptera Anatomy Ontology (http://portal.hymao.org).

Surface sculpture terminology follows Eady (1968) in most cases and Burks et al. (2013a, 2013b) in interpretations of major sculpture versus microsculpture, which are explained again here. Diminutive variant sculptural terms were avoided because of a lack of criteria for separating them from non-diminutive alternatives. “Major” surface sculpture refers to repeated sculptural patterns that interact with seta placement, not including non-repeated elements or those which are repeated only once due to bilateral symmetry. “Umbilicate-foveate” sculpture refers to rounded crater-like sculptural elements, each surrounding a setiferous pit (and thus interacting with a seta), with each fovea being much larger than its setiferous pit and spatially separated from that pit (see, e.g., Fig. 3). “Umbilicate-punctate” sculpture indicates that no sculptural element accompanies the setiferous pit (and therefore the setal pit is the “major” surface sculpture element here, e.g., T6 in Fig. 67). “Rugose” sculpture refers to a pattern of branching or wrinkling elevations that flank setiferous pits but do not fully surround them (e.g., Figs 6, 7). Rugose sculpture can coexist with umbilicate sculpture in the same area of the sclerite, in which case the rugae occur on spaces between umbilicate sculptural elements. Note that “rugose” refers to a distribution of sculptural elements, and therefore can be “irregular” or “regular” even though rugae (the elements themselves) are by definition wrinkle-like and therefore at least slightly irregular. Where both umbilicate-foveate and umbilicate-punctate sculpture are reported for the same sclerite, this should be interpreted as variable sculpture where some setiferous pits are surrounded by foveae while others are not. Under this scheme, “major” surface sculpture cannot occur in any part of the sclerite that lacks setae.

“Microsculpture” refers to repeated tiny sculptural elements that do not interact with seta placement. Microsculpture can occur on “major” sculptural elements, such as on rugae and on all surfaces of foveae. “Punctate” microsculpture refers to tiny round pits that do not bear setae. “Granulate” microsculpture refers to sculpture that is similar to that of leather or skin, with areas enclosed by tiny grooves (= sunken septa). Microsculpture can occur in areas that lack setae.

Sculptural terms for repeated sculpture that are not included in the above categories are 1) “carinae” which refers to elevations that are sharp and not branched or wrinkled but do not repeat in a way that forms a pattern (excluding repeating due to bilateral symmetry), 2) “striae” which refers to repeated elevations that are not sharp and not branched or wrinkled. These sculptural elements do not interact with setiferous pit placement, but major sculptural elements can occur between them. While alternative logic may suggest that rugose sculpture is better classed within this category, this choice was avoided because rugose sculptural patterns did apparently interact with umbilicate sculptural patterns. For the occipital carina, “crenulate” means that short carinae radiate from the occipital carina. Certain carinae may be described using the phrase “wrinkle-like,” which replaces our previous words “as a ruga,” this change being done to make the terms more clearly descriptive.

**Illustrations.** Photographs were taken using a Synoptics Ltd. system using a Leica Z16 APO microscope and a JVC KY-F75U 3-CCD camera. Source photos were stacked using Zerene Stacker version 1.04, or Auto-Montage Pro version 5.01.0005, and enhanced using Adobe Photoshop CS5 or CS6.

**Phylogenetic analysis.** A New Technology Search at initial level 95 was performed using TNT (Tree analysis using New Technology) version 1.1 (Goloboff et al. 2003, 2008). Implied weighting was used, with a default function of K = 15. Bootstrapping was performed with 1,000 replicates using the same settings but without implied weighting. *Bracalba cuneata* Dodd was used as an outgroup for the analyses (specimens OSUC 238172, OSUC 238164), chosen because of morphological similarity between *Oxyscelio* and *Bracalba*. A total of 14 out of 50 characters were used from the overall dataset (see Appendix I for characters and matrix).

## Taxonomy

### Oxyscelio

Taxon classificationAnimaliaHymenopteraPlatygastridae

Kieffer

http://zoobank.org/99E3E72E-DA88-4740-9ECB-2D03BCD1DACE

http://bioguid.osu.edu/xbiod_concepts/529

Oxyscelio Kieffer 1907: 310. Original description. Type: *Oxyscelio foveatus* Kieffer, by monotypy. See Burks et al. (2013a, b) for complete bibliography, description of the genus, and discussion of its phylogenetic position within the family.
Freniger Szabó 1956: 47. Original description. Type: *Freniger bicolor* Szabó, by monotypy and original designation. Masner 1976: 6, 19 (description, keyed). Johnson 1992: 373 (catalogued, catalog of world species). **New synonymy**

#### Internal phylogenetic relationships.

**Figure 1.**
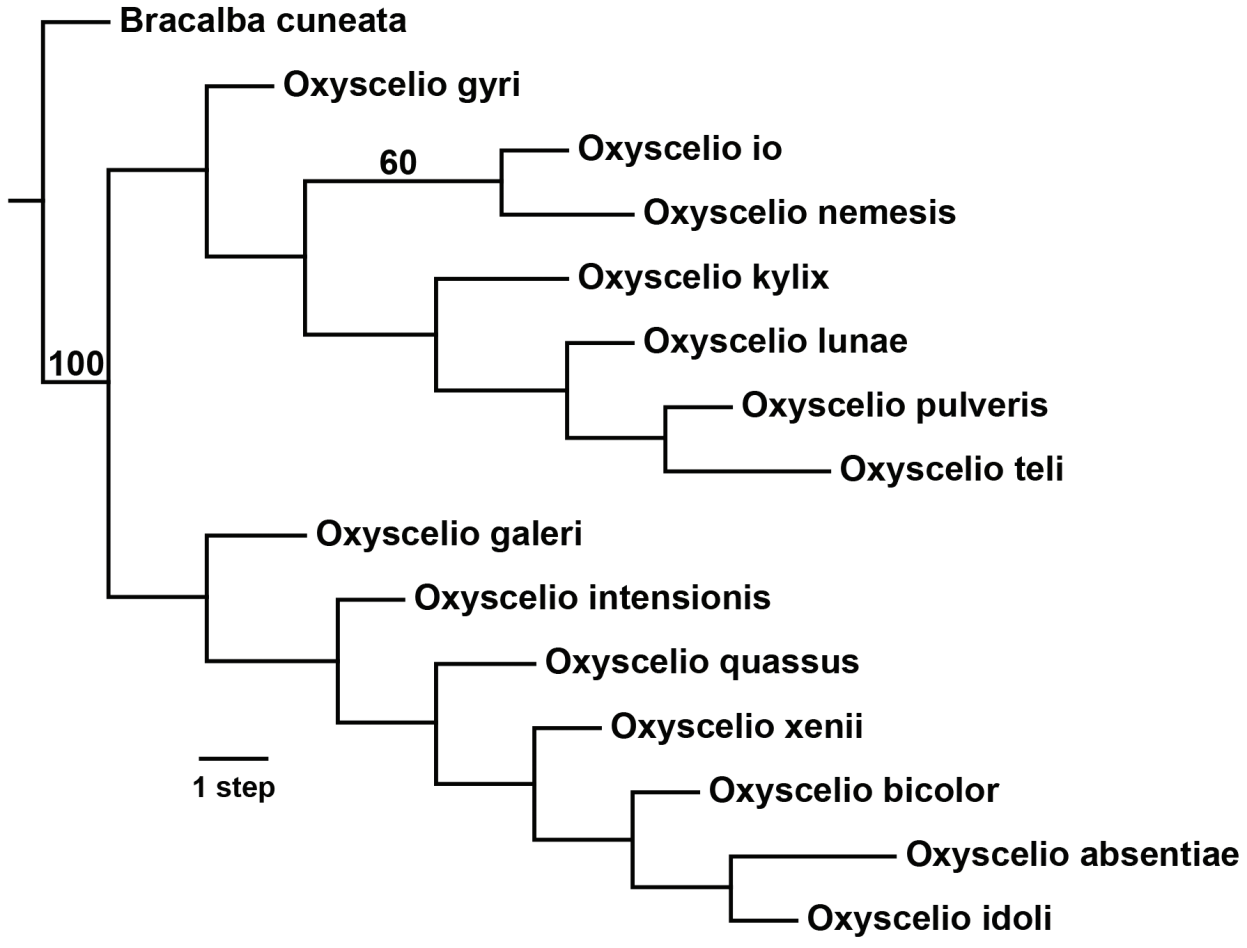
Single most parsimonious phylogram for African species of *Oxyscelio* using TNT New Technology Search with set initial level = 95, implied weighting default function K = 15. Best score = 0.74877. Bootstrap support values above 50% indicated above branches.

The phylogenetic analysis performed with a select group of characters (Fig. 1) found the *bicolor*-group and African species of the *crateris*-group to be monophyletic, with *Oxyscelio quassus* as the sister group to the *bicolor*-group and the *crateris*-group species as sister group to all other African species.

These results indicate that recognition of the genus *Freniger* (with *Freniger bicolor* as type) would make the genus *Oxyscelio* paraphyletic. Our understanding of relationships among all *Oxyscelio* species is insufficient to suggest a robust reclassification of these into monophyletic taxa. Therefore, we opt to treat *Freniger* as a junior synonym of *Oxyscelio*.

##### Species groups of African *Oxyscelio*

These groups are provided here to indicate intuitively perceived structure within the genus, and to provide an aid for identification. They are succinctly diagnosed here. Some characters are omitted situationally from species group diagnoses because those characters are variable within the group or are otherwise unhelpful for that particular group’s identification. Individual species descriptions can be consulted regarding characters omitted from these diagnoses. The only uniquely African species group is the *bicolor*-group, which is defined by a character that is unique in *Oxyscelio*. Two other African species are not placed to group, and may represent important lineages as well (see below). The more lengthy species group diagnoses for the other groups in Burks et al. (2013a, b) can be consulted for the full list of other character states that fully define the *crateris*-group and *cuculli*-group.

***Oxyscelio bicolor* species group**

**Characteristics.** Hyperoccipital carina absent or not connected to occipital carina. Hind wing vein (Sc+R) interrupted.

**Comments.** The *bicolor*-group contains species with a broadly interrupted hind wing vein (Sc+R). This feature is unique to this group within *Oxyscelio*, and previously has been used to help define the Scelionini, Nixoniini, and Sparasionini (Masner 1976)

Contains: *Oxyscelio absentiae*, *Oxyscelio bicolor*, *Oxyscelio idoli*, *Oxyscelio xenii*.

***Oxyscelio crateris* species group**

**Characteristics.** Hyperoccipital carina connected to occipital carina laterally. Hind wing vein (Sc+R) not interrupted.

**Comments.** The *crateris*-group also occurs in the Indo-Malayan realm, with species that have a slightly less pronounced “crater” on the occiput between the hyperoccipital and occipital carinae. A potential species complex within this group contains *Oxyscelio io*, *Oxyscelio nemesis*, and *Oxyscelio teli*, which are vaguely similar in the shape of the head, body, hyperoccipital carina, and occipital carina. However, that grouping was not upheld by the phylogenetic analysis and could not be characterized with any consistently definable features.

Contains: *Oxyscelio gyri*, *Oxyscelio io*, *Oxyscelio lunae*, *Oxyscelio nemesis*, *Oxyscelio pulveris*, *Oxyscelio kylix*, *Oxyscelio teli*.

***Oxyscelio cuculli* species group**

**Characteristics.** Hyperoccipital carina present as a sharp carina but not connected to occipital carina laterally. Hind wing vein (Sc+R) not interrupted.

**Comments.** The *cuculli*-group also occurs throughout the Indo-Malayan realm, with species very similar to the only known African species.

Contains: *Oxyscelio galeri*.

**Species not placed to group**

**Included species**: *Oxyscelio intensionis*, *Oxyscelio quassus*.

**Comments.** There is some general resemblance between *Oxyscelio intensionis* and the Australian *aciculae*-group, but members of that group do not have a setose metasomal depression. *Oxyscelio quassus* has a setose metasomal depression, but lacks the important features of other species groups, resembling the *bicolor*-group but having a complete hind wing vein. The Indo-Malayan and Australian *dasymesos*-group differs from *Oxyscelio intensionis* in occipital sculpture and in having sharp projections from the corners of T7. The *dasymesos*-group differs from *Oxyscelio quassus* in having a complete mesoscutal median carina, and in having a very different (short and stout) body shape.

##### Key to African species of *Oxyscelio*

**Table:** 

1	Hind wing vein (Sc+R) incomplete, broadly interrupted between base and apex (Fig. 4, 77). (*bicolor s*pecies group)	**2**
–	Hind wing vein (Sc+R) complete (Figs 55, 66–67)	**5**
2	Metasomal depression setose (Figs 10, 32–33, 56–57, 65, 75–76)	**3**
–	Metasomal depression not setose (Figs 21, 49)	**4**
3	T1 without carinae between midlobe and lateral areas (Figs 75–77)	***Oxyscelio xenii* Burks, sp. n.**
–	T1 with one or more longitudinal carinae between midlobe and lateral areas (Figs 9–10)	***Oxyscelio bicolor* (Szabó)**
4	Metascutellum much broader than long (Fig. 3); female T1 with very long anterior horn (Figs 3, 5)	***Oxyscelio absentiae* Burks, sp. n.**
–	Metascutellum about as broad as long (Fig. 23); female T1 without anterior horn (Figs 23, 25)	***Oxyscelio idoli* Burks, sp. n.**
5	Metascutellum triangular, acuminate posteriorly (Fig. 13). (*cuculli* group)	***Oxyscelio galeri* Burks, sp. n.**
–	Metascutellum not triangular, not acuminate apically (Figs 17, 32–33, 41, 47, 49, 56–57, 59, 65, 69)	**6**
6	Metasomal depression setose (Figs 32–33, 56–57, 65)	**7**
–	Metasomal depression not setose (Fig. 21) (*crateris* group, in part)	**9**
7	Occipital carina medially flat (Fig. 53) (*crateris* group, in part)	***Oxyscelio nemesis* Burks, sp. n.**
–	Occipital carina medially arched (Fig. 29)	**8**
8	Hyperoccipital carina indicated by a set of complete wrinkle-like carinae (Fig. 29)	***Oxyscelio intensionis* Burks, sp. n.**
–	Hyperoccipital carina absent (Figs 62–63)	***Oxyscelio quassus* Burks, sp. n.**
9	Mesoscutellum with some granulate sculpture (Figs 35, 47, 59)	**10**
–	Mesoscutellum without granulate sculpture (Figs 17, 41, 69)	**12**
10	Frontal depression with median longitudinal carina-like elevation arising from interantennal process (Fig. 36)	***Oxyscelio io* Burks, sp. n.**
–	Frontal depression without median longitudinal carina-like elevation (Figs 48, 60)	**11**
11	Head and mesosomal dorsum with sharp carinae and less extensive granulate sculpture (Figs 46–47)	***Oxyscelio lunae* Burks, sp. n.**
–	Head and mesosomal dorsum with weak carinae and more extensive granulate sculpture (Figs 58–59)	***Oxyscelio pulveris* Burks, sp. n.**
12	Occipital carina medially flat (Fig. 69)	***Oxyscelio teli* Burks, sp. n.**
–	Occipital carina arched or sinuate medially (Figs 17, 41).	**13**
13	Occipital carina with sharp lateral corners, connected to hyperoccipital carina laterally (Fig. 41)	***Oxyscelio kylix* Burks, sp. n.**
–	Occipital carina without lateral corners, not connected to hyperoccipital carina laterally (Fig. 17)	***Oxyscelio gyri* Burks, sp. n.**

**Figures 2–5.**
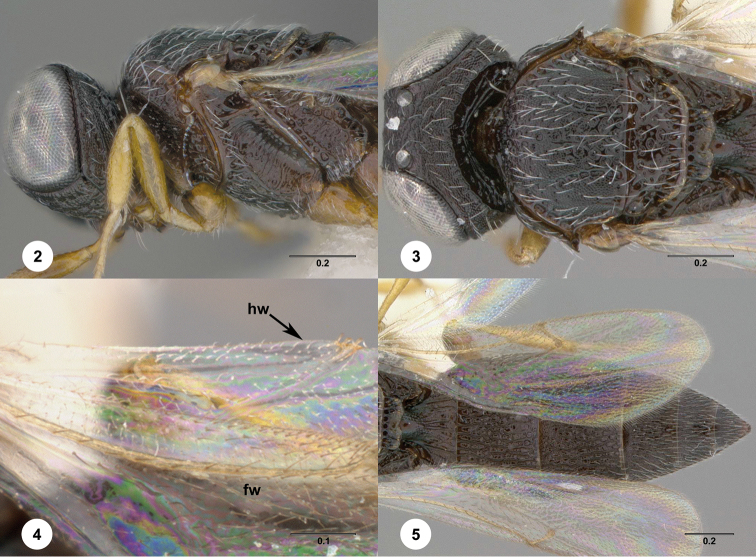
*Oxyscelio absentiae* sp. n., paratype female (OSUC 369414) **2** Head and mesosoma, lateral view **3** Head and mesosoma, dorsal view **4** Hind wing, dorsal view (fw = fore wing, hw = hind wing) **5** Metasoma, dorsal view. Morphbank^14^

**Figures 6–11.**
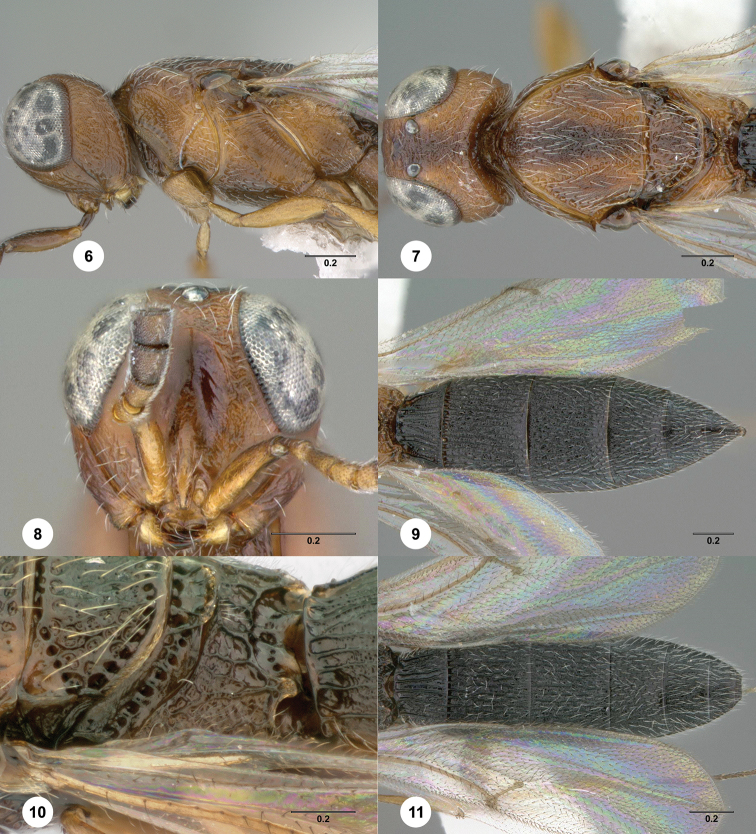
*Oxyscelio bicolor* (Szabó), female (OSUC 369418) **6** Head and mesosoma, lateral view **7** Head and mesosoma, dorsal view **8** Head, anterior view **9** Metasoma, dorsal view. Female (OSUC 369371) **10** Propodeum, dorsolateral view. Male (OSUC 369427) **11** Metasoma, dorsal view. Morphbank^15^

**Figures 12–15.**
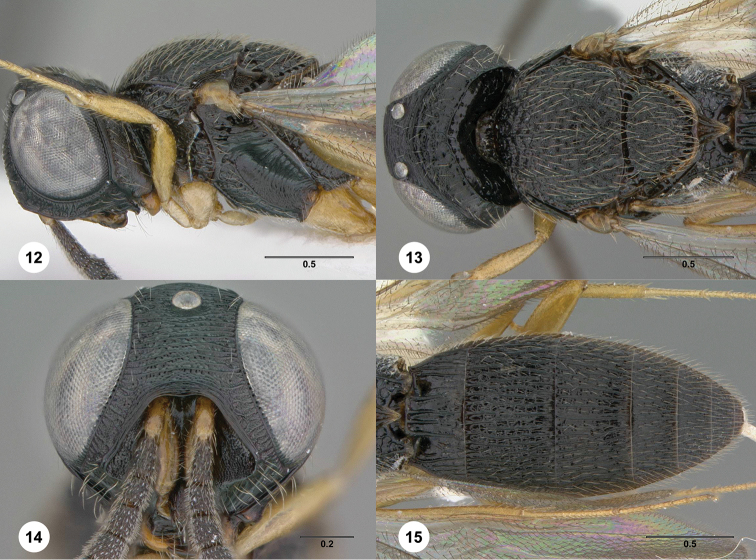
*Oxyscelio galeri* sp. n., paratype male (OSUC 369355) **12** Head and mesosoma, lateral view **13** Head and mesosoma, dorsal view **14** Head, anterior view **15** Metasoma, dorsal view. Morphbank^16^

**Figures 16–21.**
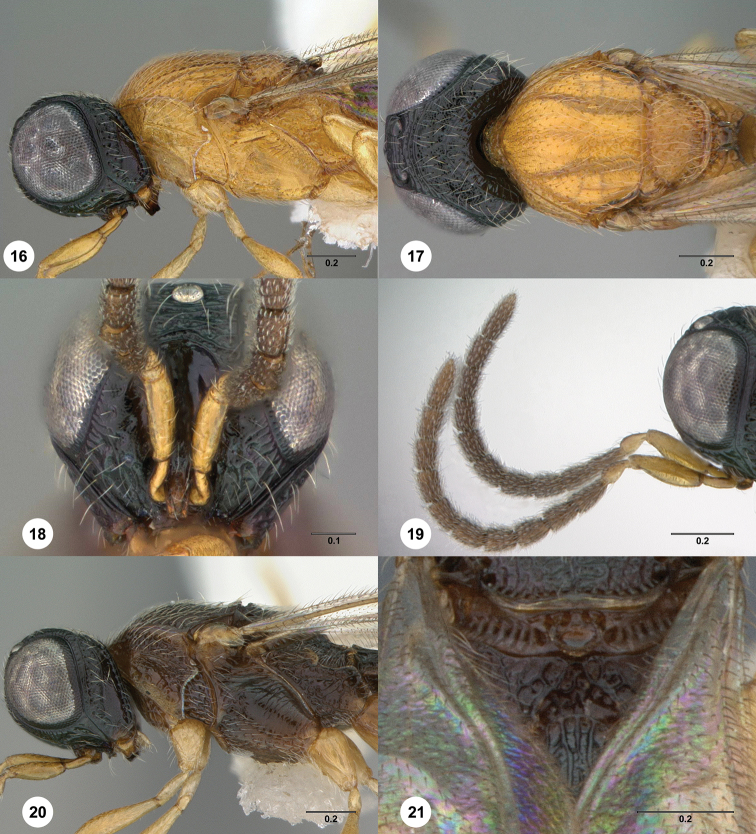
*Oxyscelio gyri* sp. n., holotype female (OSUC 369372) **16** Head and mesosoma, lateral view **17** Head and mesosoma, dorsal view Paratype male (OSUC 369374) **18** Head, anterior view **19** Antenna **20** Mesosoma, lateral view **21** Propodeum, posterior view. Morphbank^17^

**Figures 22–27.**
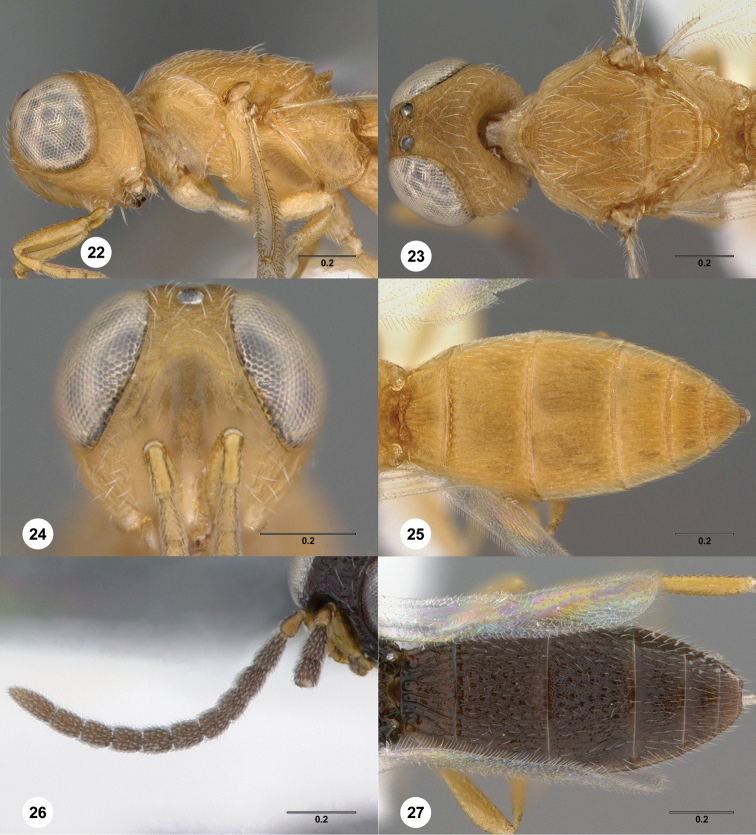
*Oxyscelio idoli* sp. n., holotype female (OSUC 369367) **22** Head and mesosoma, lateral view **23** Head and mesosoma, dorsal view **24** Head, anterior view **25** Metasoma, dorsal view. Paratype male (OSUC 369368) **26** Antenna **27** Metasoma, dorsal view. Morphbank^18^

**Figures 28–33.**
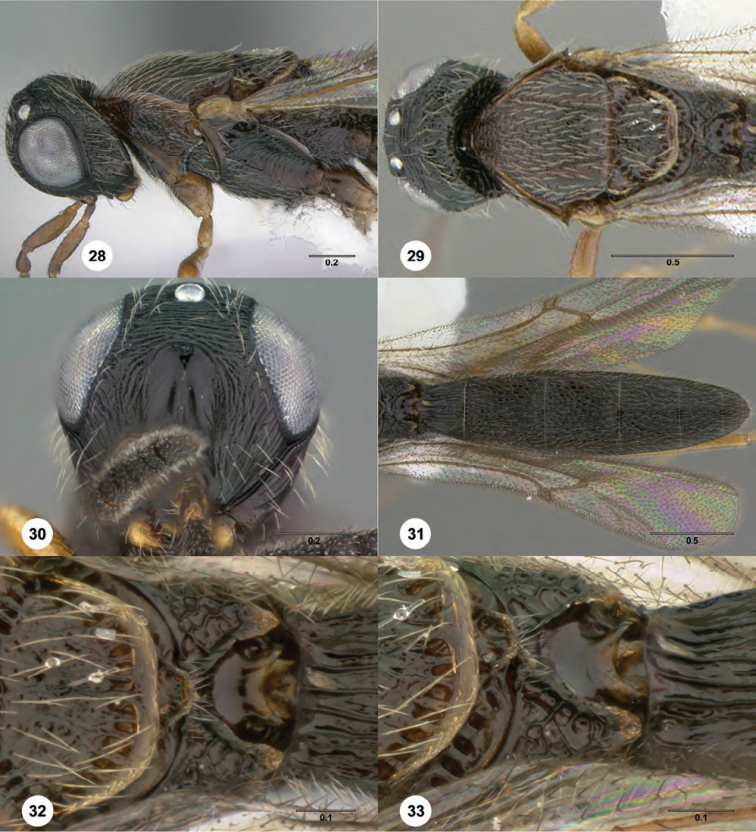
*Oxyscelio intensionis* sp. n., holotype male (OSUC 369369) **28** Head and mesosoma, lateral view **29** Head and mesosoma, dorsal view **30** Head, anterior view **31** Metasoma, dorsal view **32** Propodeum, dorsal view **33** Propodeum, dorsolateral view. Morphbank^19^

**Figures 34–39.**
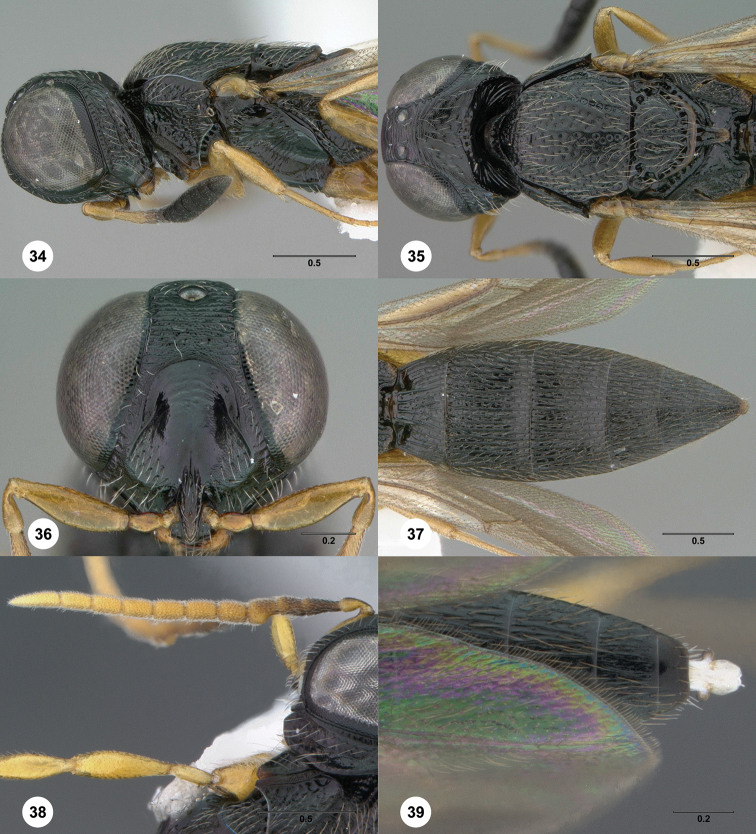
*Oxyscelio io* sp. n., holotype female (OSUC 369403) **34** Head and mesosoma, lateral view **35** Head and mesosoma, dorsal view **36** Head, anterior view **37** Metasoma, dorsal view. Paratype male (OSUC 470506) **38** Antenna **39** Metasomal apex, dorsal view. Morphbank^20^

**Figures 40–45.**
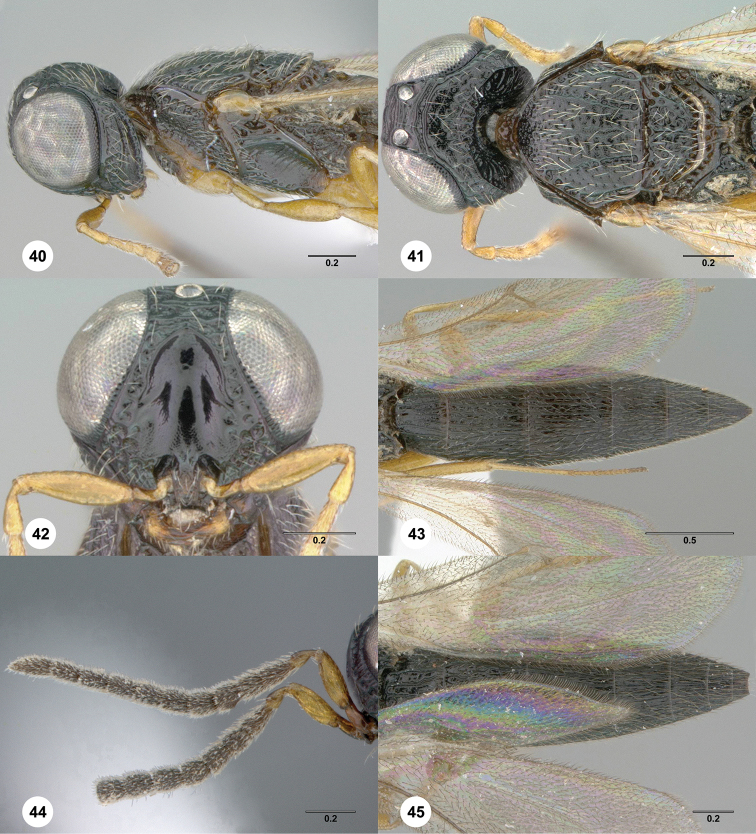
*Oxyscelio kylix* sp. n., holotype female (OSUC 369399) **40** Head and mesosoma, lateral view **41** Head and mesosoma, dorsal view **42** Head, anterior view **43** Metasoma, dorsal view. Paratype male (OSUC 369389) **44** Antenna **45** Metasoma, dorsal view. Morphbank^21^

**Figures 46–51.**
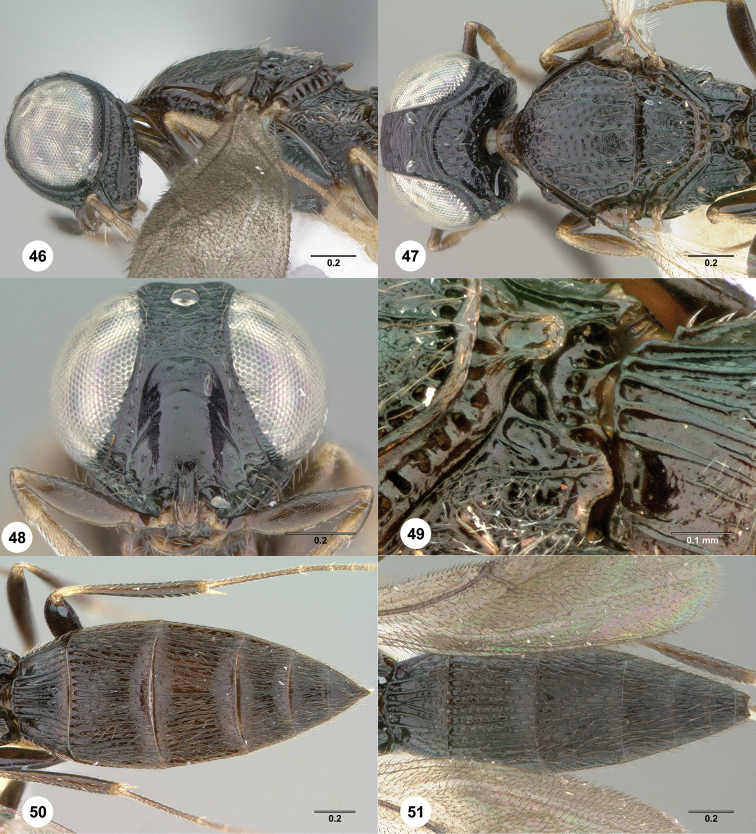
*Oxyscelio lunae* sp. n., paratype female (OSUC 369409) **46** Head and mesosoma, lateral view **47** Head and mesosoma, dorsal view **48** Head, anterior view **49** Metasoma, dorsal view **50** Propodeum, dorsolateral view. Paratype Male (OSUC 369404) **51** Metasoma, dorsal view. Morphbank^22^

**Figures 52–57.**
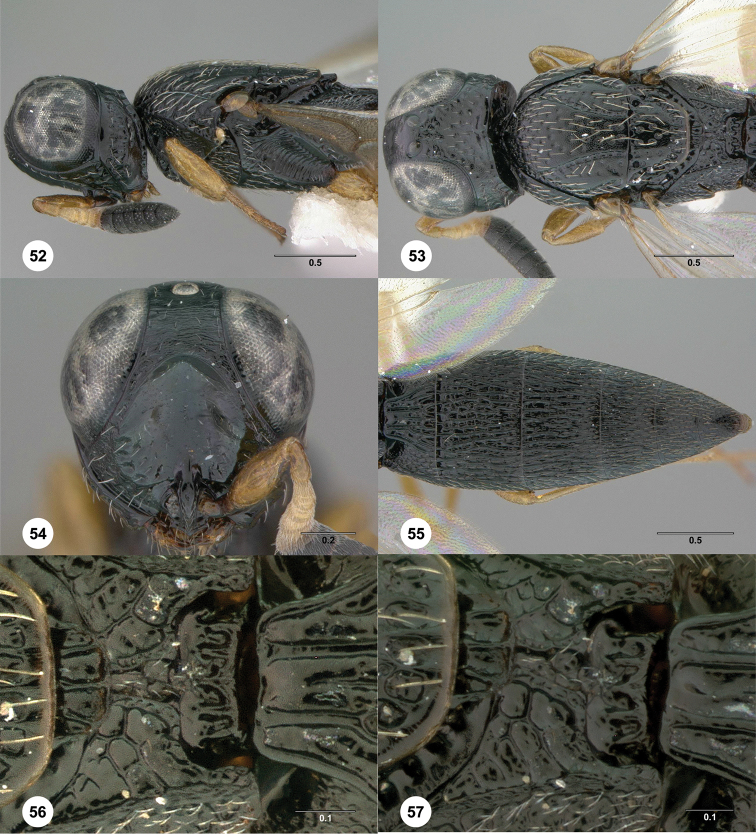
*Oxyscelio nemesis* sp. n., holotype female (OSUC 369379) **52** Head and mesosoma, lateral view **53** Head and mesosoma, dorsal view **54** Head, anterior view **55** Metasoma, dorsal view **56** Propodeum, dorsal view **57** Propodeum, dorsolateral view. Morphbank^23^

**Figures 58–61.**
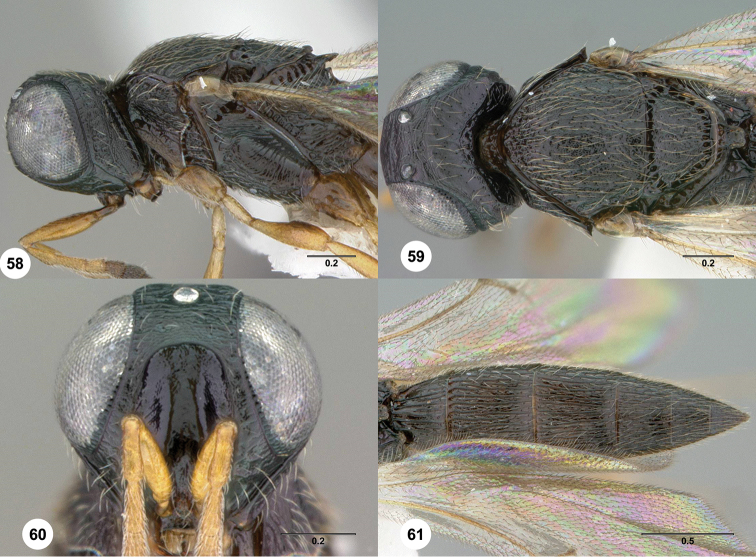
*Oxyscelio pulveris* sp. n., holotype female (OSUC 369388) **58** Head and mesosoma, lateral view **59** Head and mesosoma, dorsal view **60** Head, anterior view **61** Metasoma, dorsal view. Morphbank^24^

**Figures 62–67.**
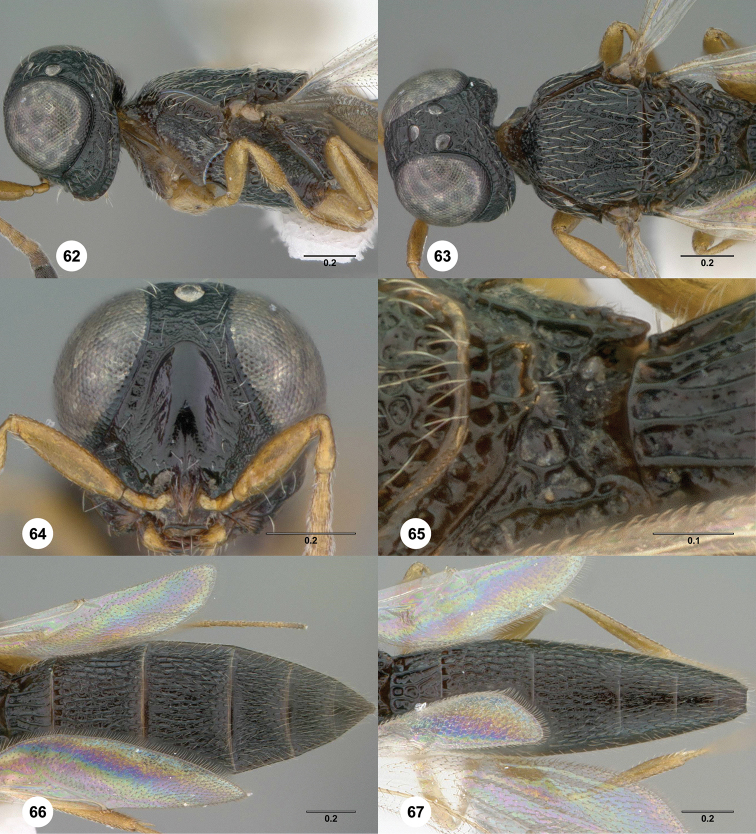
*Oxyscelio quassus* sp. n., holotype female (OSUC 369398) **62** Head and mesosoma, lateral view **63** Head and mesosoma, dorsal view **64** Head, anterior view **65** Propodeum, dorsolateral view **66** Metasoma, dorsal view. Paratype male (OSUC 369402) **67** Metasoma, dorsal view Morphbank^25^

**Figures 68–71.**
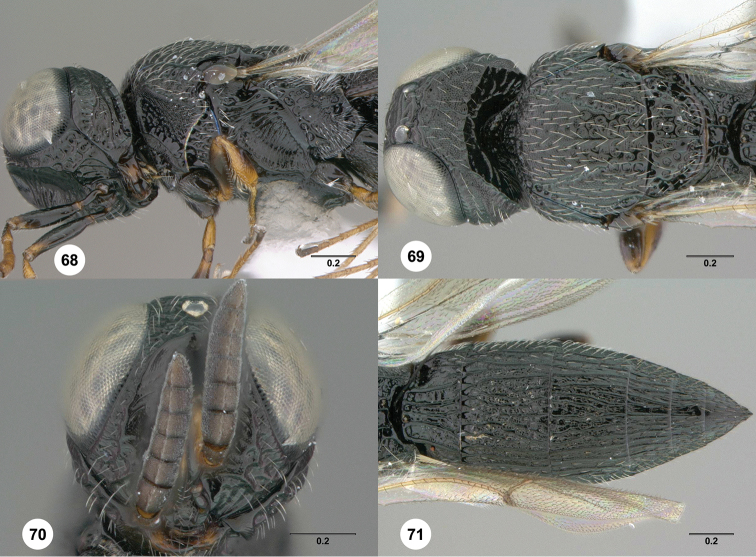
*Oxyscelio teli* sp. n., holotype female (OSUC 381658) **68** Head and mesosoma, lateral view **69** Head and mesosoma, dorsal view **70** Head, anterior view **71** Metasoma, dorsal view. Morphbank^26^

**Figures 72–77.**
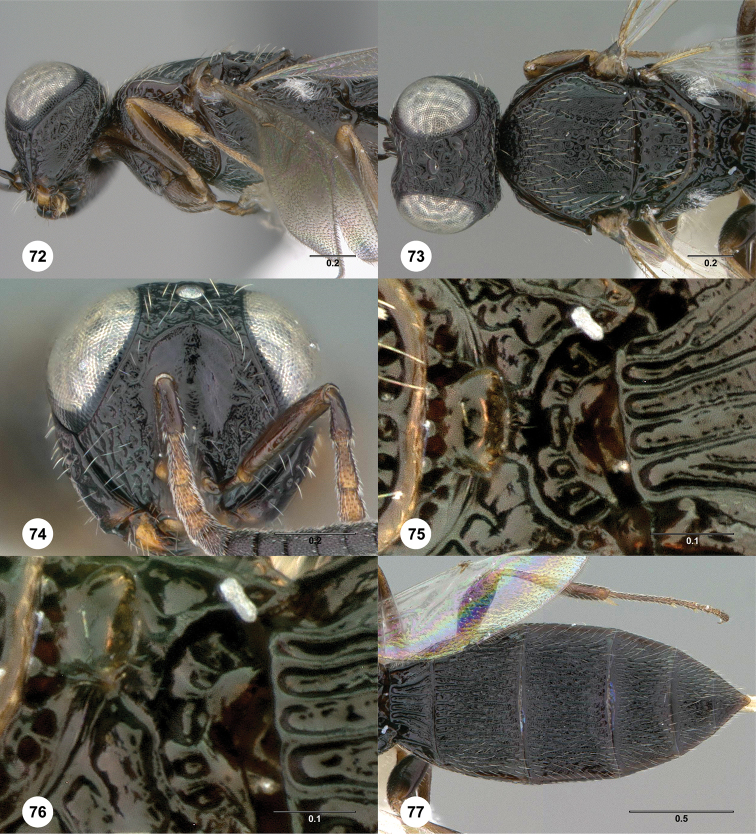
*Oxyscelio xenii* sp. n., holotype female (OSUC 369376) **72** Head and mesosoma, lateral view **73** Head and mesosoma, dorsal view **74** Head, anterior view **75** Propodeum, dorsal view **76** Propodeum, dorsolateral view **77** Metasoma, dorsal view. Morphbank^27^

### Species descriptions

#### Oxyscelio absentiae

Taxon classificationAnimaliaHymenopteraPlatygastridae

Burks sp. n.

http://zoobank.org/5C78286D-3D78-4825-9EBA-81DCABC13E66

http://bioguid.osu.edu/xbiod_concepts/309292

Figures 2–5 ; Morphbank ^14^

##### Description.

*Female*. Body length 2.5–2.65 mm (n = 4).

Radicle color: same as scape. A4: broader than long. A5: broader than long. Upper frons: not hood-like. Frontal depression sculpture: with 3 or more broadly interrupted transverse carinae; with 2–4 complete transverse carinae. Median longitudinal elevation in frontal depression: absent. Major sculpture of gena anteroventrally: rugose; umbilicate-punctate. Major sculpture of gena posteroventrally: umbilicate-punctate. Microsculpture of gena anteroventrally: granulate. Microsculpture of gena posteroventrally: granulate. Hyperoccipital carina: not indicated medially. Median carina extending posteriorly from hyperoccipital carina: absent. Lateral connection between hyperoccipital and occipital carinae: absent. Area between vertex and occipital carina: umbilicate-foveate; umbilicate-punctate. Occipital carina medially: uniformly rounded. Lateral corners of occipital carina: absent.

Mesoscutum anteriorly: steep. Mesoscutal median carina: absent or incomplete. Major sculpture of mesoscutal midlobe anteriorly: umbilicate-foveate. Major sculpture of mesoscutal midlobe posteriorly: umbilicate-foveate. Microsculpture of mesoscutal midlobe anteriorly: granulate. Microsculpture of mesoscutal midlobe posteriorly: absent. Major sculpture of mesoscutellum: umbilicate-foveate. Microsculpture of mesoscutellum medially: punctate. Microsculpture of mesoscutellum laterally: punctate. Number of carinae crossing femoral depression: 4 or more. Mesepimeral sulcus pits: more than 5. Setae along anterior limit of femoral depression: arising from rows of foveae. Metascutellum dorsally: flat or convex. Metascutellar sculpture centrally: with longitudinal carinae. Metascutellar apex: deeply emarginate; shallowly emarginate. Metapleuron above ventral metapleural area: foveate or rugose. Lateral propodeal carinae antero-medially: strongly diverging. Metasomal depression setae: absent. Anterior areoles of metasomal depression: absent. Anterior longitudinal carinae in metasomal depression: absent. Postmarginal vein: present. Fore wing apex: reaching middle of T5. Hind wing vein (Sc+R): interrupted.

Carinae between T1 midlobe and T1 lateral carina: present. T1 midlobe: obscured by other raised sculpture. T1: with long anterior bulge that reaches metascutellum. T6: longer than broad; as long as broad. Metasomal apex: rounded. Major sculpture of T6: umbilicate-punctate. Microsculpture of T6: absent; granulate.

##### Diagnosis.

Both sexes: Hyperoccipital carina absent. Gena with granulate sculpture anteroventrally and posteroventrally. Mesoscutellum without granulate sculpture. Metascutellum much broader than long. Metasomal depression not setose, without median carina; lateral propodeal carinae strongly diverging. Hind wing Sc+R interrupted. T1 with carinae between midlobe and lateral carina. Female: A4 broader than long; T1 midlobe with strong anterior horn.

##### Etymology.

Latin noun, genitive case, meaning “absence.”

##### Link to distribution map.

[http://hol.osu.edu/map-full.html?id=309292]

##### Material examined.

Holotype, female: **SOMALIA**: Mogadishu, Shabelle (Shabelli) Valley, Afgooye (Afgoi), 1.II–15.II.1977, malaise trap, F. Bin, OSUC 369416 (deposited in CNCI). *Paratypes*: **SOMALIA**: 3 females, OSUC 369414–369415, 369417 (CNCI).

#### Oxyscelio bicolor

Taxon classificationAnimaliaHymenopteraPlatygastridae

(Szabó) comb. n.

http://zoobank.org/FE1CAEFA-4FFD-4ADC-B7A4-55D1F5FA55A6

http://bioguid.osu.edu/xbiod_concepts/4310

Figures 6–11 ; Morphbank ^15^

Freniger bicolor Szabó 1956: 48 (original description); Masner 1976: 20 (type information).

##### Description.

*Female*. Body length 3.15–3.55 mm (n = 5).

Radicle color: same as scape; darker than scape. A4: longer than broad. A5: broader than long. Upper frons: not hood-like. Frontal depression sculpture: with 1–2 broadly interrupted transverse carinae. Median longitudinal elevation in frontal depression: absent. Major sculpture of gena anteroventrally: umbilicate-foveate; rugose. Major sculpture of gena posteroventrally: rugose; umbilicate-punctate. Microsculpture of gena anteroventrally: granulate. Microsculpture of gena posteroventrally: granulate. Hyperoccipital carina: wrinkle-like. Median carina extending posteriorly from hyperoccipital carina: absent. Lateral connection between hyperoccipital and occipital carinae: absent. Area between vertex and occipital carina: umbilicate-foveate; rugose. Occipital carina medially: uniformly rounded. Lateral corners of occipital carina: absent.

Mesoscutum anteriorly: not steep. Mesoscutal median carina: absent or incomplete. Major sculpture of mesoscutal midlobe anteriorly: umbilicate-foveate. Major sculpture of mesoscutal midlobe posteriorly: umbilicate-foveate. Microsculpture of mesoscutal midlobe anteriorly: granulate. Microsculpture of mesoscutal midlobe posteriorly: absent; granulate. Major sculpture of mesoscutellum: umbilicate-foveate; obliquely rugose. Microsculpture of mesoscutellum medially: absent. Microsculpture of mesoscutellum laterally: absent. Number of carinae crossing femoral depression: 4 or more. Mesepimeral sulcus pits: more than 5. Setae along anterior limit of femoral depression: arising from rows of foveae. Metascutellum dorsally: concave. Metascutellar sculpture centrally: smooth; rugose. Metascutellar apex: convex or straight. Metapleuron above ventral metapleural area: foveate or rugose. Lateral propodeal carinae antero-medially: weakly diverging. Metasomal depression setae: present. Anterior areoles of metasomal depression: one or more areoles present. Anterior longitudinal carinae in metasomal depression: absent. Postmarginal vein: present. Fore wing apex: reaching apex of T6; reaching beyond T6. Hind wing vein (Sc+R): interrupted.

Carinae between T1 midlobe and T1 lateral carina: present. T1 midlobe: with 6 or more longitudinal carinae. T1: without anterior bulge. T6: broader than long. Metasomal apex: rounded. Major sculpture of T6: umbilicate-punctate. Microsculpture of T6: granulate.

*Male*. Body length 3.15–3.4 mm (n = 7). A5 tyloid: carina-like, not expanded. A11: longer than broad. T1 midlobe: with 5 longitudinal carinae. Metasomal apex: with acuminate lateral corners.

##### Diagnosis.

Both sexes: Hyperoccipital carina wrinkle-like, not connected to occipital carina laterally or medially. Gena with granulate sculpture anteroventrally and posteroventrally. Mesoscutellum without granulate sculpture; without punctate sculpture between foveae. Metasomal depression setose, without median carina; lateral propodeal carinae weakly diverging. Hind wing Sc+R interrupted. T1 with carinae between midlobe and lateral carina. Female: A4 longer than broad; T1 without anterior horn.

##### Link to distribution map.

[http://hol.osu.edu/map-full.html?id=4310]

##### Material examined.

Holotype, female: **TANZANIA**: Arusha Reg., Upper Arusha (Arusha-Ju), X–1905, Katona, Hym.Typ.No. 9553, Mus.Budapest (deposited in HNHM). Other material: (4 females, 8 males) **KENYA**: 3 females, 7 males, OSUC 369418, 369425–369433 (CNCI). **TANZANIA**: 1 female, 1 male, OSUC 369370–369371 (CNCI).

##### Comments.

*Freniger* Szabó represents an unusual species group of African *Oxyscelio*, with a broadly interrupted hind wing vein (Sc+R). The metasomal depression setae in this and some other African *Oxyscelio* are rarely found in species outside Africa – only in the two Asian and single Australian species of the *dasymesos* group.

#### Oxyscelio galeri

Taxon classificationAnimaliaHymenopteraPlatygastridae

Burks sp. n.

http://zoobank.org/51C5ECA4-5C4D-402E-A7A4-8F2E7E4995F3

http://bioguid.osu.edu/xbiod_concepts/309293

Figures 12–15 ; Morphbank ^16^

##### Description.

*Male*. Body length 3.95–4.1 mm (n = 5).

Radicle color: same as scape. A5 tyloid: carina-like, not expanded. A11: longer than broad. Upper frons: hood-like, protruding over pedicel when antenna at rest. Frontal depression sculpture: without transverse or oblique carinae below submedian carina. Median longitudinal elevation in frontal depression: absent. Major sculpture of gena anteroventrally: umbilicate-foveate. Major sculpture of gena posteroventrally: umbilicate-punctate. Microsculpture of gena anteroventrally: granulate. Microsculpture of gena posteroventrally: granulate. Hyperoccipital carina: complete as a sharp carina. Median carina extending posteriorly from hyperoccipital carina: absent. Lateral connection between hyperoccipital and occipital carinae: absent. Area between vertex and occipital carina: umbilicate-foveate; rugose. Occipital carina medially: sinuate with a more strongly arched median portion. Lateral corners of occipital carina: absent.

Mesoscutum anteriorly: not steep. Mesoscutal median carina: present and complete. Major sculpture of mesoscutal midlobe anteriorly: umbilicate-foveate. Major sculpture of mesoscutal midlobe posteriorly: umbilicate-foveate; transversely rugose; obliquely rugose. Microsculpture of mesoscutal midlobe anteriorly: granulate. Microsculpture of mesoscutal midlobe posteriorly: granulate. Major sculpture of mesoscutellum: umbilicate-foveate; obliquely rugose. Microsculpture of mesoscutellum medially: granulate. Microsculpture of mesoscutellum laterally: granulate. Number of carinae crossing femoral depression: 4 or more. Mesepimeral sulcus pits: more than 5. Setae along anterior limit of femoral depression: arising from rows of foveae. Metascutellum dorsally: concave. Metascutellar sculpture centrally: with longitudinal carinae. Metascutellar apex: sharply acuminate. Metapleuron above ventral metapleural area: foveate or rugose. Lateral propodeal carinae antero-medially: strongly diverging. Metasomal depression setae: absent. Anterior areoles of metasomal depression: absent. Anterior longitudinal carinae in metasomal depression: absent. Postmarginal vein: present. Hind wing vein (Sc+R): not interrupted.

Carinae between T1 midlobe and T1 lateral carina: absent. T1 midlobe: with 4 longitudinal carinae. Metasomal apex: with no distinct corners.

##### Diagnosis.

Both sexes: Frontal depression forming hood-like structure (deep and with strongly protruding submedian carina that overhangs pedicels). Hyperoccipital carina present and sharp, not connected to occipital carina laterally; median carina between hyperoccipital carina and occipital carina absent. Gena with granulate sculpture anteroventrally and posteroventrally. Mesoscutellum with granulate sculpture. Metascutellum acuminate apically. Metasomal depression without median carina; lateral propodeal carinae strongly diverging. Hind wing Sc+R complete.

##### Etymology.

Latin noun, genitive case, referring to a kind of helmet.

##### Link to distribution map.

[http://hol.osu.edu/map-full.html?id=309293]

##### Material examined.

Holotype, female: **CAMEROON**: Centre Prov., Mbalmayo, VII–1993, malaise trap, P. Eggleton, OSUC 369356 (deposited in BMNH). *Paratypes*: ﻿﻿**CAMEROON**: 5 males, OSUC 369353–369355 (CNCI), 369357–369358 (BMNH).

##### Comments.

The other members of the *cuculli* group are widespread in Asia, including China and India. *Oxyscelio galeri* is distinct within this group due to its acuminate metascutellum.

#### Oxyscelio gyri

Taxon classificationAnimaliaHymenopteraPlatygastridae

Burks sp. n.

http://zoobank.org/43C8DD10-DDEA-40DE-AAA5-2CB62ED3FE09

http://bioguid.osu.edu/xbiod_concepts/309294

Figures 16–21 ; Morphbank ^17^

##### Description.

*Female*. Body length 3.35 mm (n = 1).

Radicle color: same as scape. A4: longer than broad. A5: longer than broad; as long as broad. Upper frons: not hood-like. Frontal depression sculpture: with 2–4 complete transverse carinae. Median longitudinal elevation in frontal depression: absent. Major sculpture of gena anteroventrally: umbilicate-foveate. Major sculpture of gena posteroventrally: absent. Microsculpture of gena anteroventrally: absent. Microsculpture of gena posteroventrally: granulate. Hyperoccipital carina: complete as a sharp carina. Median carina extending posteriorly from hyperoccipital carina: absent. Lateral connection between hyperoccipital and occipital carinae: absent. Area between vertex and occipital carina: rugose; umbilicate-punctate. Occipital carina medially: uniformly rounded. Lateral corners of occipital carina: absent.

Mesoscutum anteriorly: not steep. Mesoscutal median carina: present and complete; absent or incomplete. Major sculpture of mesoscutal midlobe anteriorly: umbilicate-foveate; umbilicate-punctate. Major sculpture of mesoscutal midlobe posteriorly: umbilicate-foveate; obliquely rugose. Microsculpture of mesoscutal midlobe anteriorly: granulate. Microsculpture of mesoscutal midlobe posteriorly: absent. Major sculpture of mesoscutellum: umbilicate-foveate; obliquely rugose. Microsculpture of mesoscutellum medially: absent. Microsculpture of mesoscutellum laterally: absent. Number of carinae crossing femoral depression: 4 or more. Mesepimeral sulcus pits: more than 5. Setae along anterior limit of femoral depression: arising from rows of foveae. Metascutellum dorsally: concave. Metascutellar sculpture centrally: smooth. Metascutellar apex: convex or straight. Metapleuron above ventral metapleural area: crossed by carinae. Lateral propodeal carinae antero-medially: strongly diverging; weakly diverging. Metasomal depression setae: absent. Anterior areoles of metasomal depression: absent. Anterior longitudinal carinae in metasomal depression: absent. Postmarginal vein: present. Fore wing apex: reaching middle of T6. Hind wing vein (Sc+R): not interrupted.

Carinae between T1 midlobe and T1 lateral carina: present. T1 midlobe: with 5 longitudinal carinae; with 6 or more longitudinal carinae. T1: without anterior bulge. T6: broader than long; as long as broad. Metasomal apex: rounded. Major sculpture of T6: umbilicate-punctate; longitudinally striate or rugose. Microsculpture of T6: granulate.

*Male*. Body length 3.2 mm (n = 1). A5 tyloid: carina-like, not expanded. A11: longer than broad. T1 midlobe: with 5 longitudinal carinae. Metasomal apex: with acuminate lateral corners.

##### Diagnosis.

Both sexes: Hyperoccipital carina present and sharp, not connected to occipital carina laterally; median carina between hyperoccipital and occipital carinae absent. Gena with granulate sculpture posteroventrally but not anteroventrally. Mesoscutellum without granulate sculpture. Metasomal depression without median carina; lateral propodeal carinae strongly or weakly diverging. Hind wing Sc+R vein complete. Female: A4 longer than broad.

##### Etymology.

Latin noun, genitive case, meaning “circle.”

##### Link to distribution map.

[http://hol.osu.edu/map-full.html?id=309294]

##### Material examined.

Holotype, female: **TANZANIA**: Tanga Reg., hills, Amani, 23.VI–24.VII.2001, D. Quicke, OSUC 369372 (deposited in BMNH). *Paratypes*: **TANZANIA**: 1 female, 1 male, OSUC 369373, 369374 (BMNH).

#### Oxyscelio idoli

Taxon classificationAnimaliaHymenopteraPlatygastridae

Burks sp. n.

http://zoobank.org/97BEFB0C-F785-48A3-B660-72AF7AE6B20A

http://bioguid.osu.edu/xbiod_concepts/309295

Figures 22–27 ; Morphbank ^18^

##### Description.

*Female*. Body length 2.55–2.6 mm (n = 2).

Radicle color: same as scape. A4: longer than broad; as long as broad. A5: broader than long. Upper frons: not hood-like. Frontal depression sculpture: with 2–4 complete transverse carinae; with 1–2 broadly interrupted transverse carinae. Median longitudinal elevation in frontal depression: absent. Major sculpture of gena anteroventrally: rugose; umbilicate-punctate. Major sculpture of gena posteroventrally: umbilicate-foveate; rugose. Microsculpture of gena anteroventrally: granulate. Microsculpture of gena posteroventrally: granulate. Hyperoccipital carina: wrinkle-like. Median carina extending posteriorly from hyperoccipital carina: absent. Lateral connection between hyperoccipital and occipital carinae: absent. Area between vertex and occipital carina: rugose; umbilicate-punctate. Occipital carina medially: uniformly rounded. Lateral corners of occipital carina: absent.

Mesoscutum anteriorly: not steep. Mesoscutal median carina: present and complete; absent or incomplete. Major sculpture of mesoscutal midlobe anteriorly: umbilicate-foveate; umbilicate-punctate. Major sculpture of mesoscutal midlobe posteriorly: umbilicate-foveate. Microsculpture of mesoscutal midlobe anteriorly: granulate. Microsculpture of mesoscutal midlobe posteriorly: absent; granulate. Major sculpture of mesoscutellum: umbilicate-foveate; obliquely rugose. Microsculpture of mesoscutellum medially: absent. Microsculpture of mesoscutellum laterally: absent. Number of carinae crossing femoral depression: 4 or more. Mesepimeral sulcus pits: more than 5. Setae along anterior limit of femoral depression: arising from rows of foveae. Metascutellum dorsally: concave. Metascutellar sculpture centrally: smooth. Metascutellar apex: convex or straight; shallowly emarginate. Metapleuron above ventral metapleural area: crossed by carinae. Lateral propodeal carinae antero-medially: weakly diverging. Metasomal depression setae: absent. Anterior areoles of metasomal depression: one or more areoles present. Anterior longitudinal carinae in metasomal depression: absent. Postmarginal vein: present. Fore wing apex: reaching beyond T6. Hind wing vein (Sc+R): interrupted.

Carinae between T1 midlobe and T1 lateral carina: present. T1 midlobe: with 6 or more longitudinal carinae. T1: without anterior bulge. T6: broader than long. Metasomal apex: rounded. Major sculpture of T6: umbilicate-punctate. Microsculpture of T6: absent.

*Male*. Body length 2.4 mm (n = 1). A5 tyloid: carina-like, not expanded. A11: longer than broad. T1 midlobe: with 4 longitudinal carinae. Metasomal apex: with acuminate lateral corners.

##### Diagnosis.

Both sexes: Hyperoccipital carina wrinkle-like. Gena with granulate sculpture anteroventrally and posteroventrally. Mesoscutellum without granulate sculpture. Metascutellum about as broad as long. Metasomal depression not setose; lateral propodeal carinae weakly diverging. Hind wing Sc+R interrupted. Female: T1 midlobe without anterior horn.

##### Etymology.

Latin noun, genitive case, meaning “ghost.”

##### Link to distribution map.

[http://hol.osu.edu/map-full.html?id=309295]

##### Material examined.

Holotype, female: **TANZANIA**: Tanga Reg., Muheza Dist., canopy, Kwangumi Forest Reserve, 04°57'S 38°44'E, 9.XI.1995, fogging, OSUC 369367 (deposited in BMNH). *Paratypes*: **TANZANIA**: 1 female, 1 male, OSUC 369366, 369368 (BMNH).

#### Oxyscelio intensionis

Taxon classificationAnimaliaHymenopteraPlatygastridae

Burks sp. n.

http://zoobank.org/828EFF7A-C702-46CA-8E73-C523925A3ABC

http://bioguid.osu.edu/xbiod_concepts/309296

Figures 28–33 ; Morphbank ^19^

##### Description.

*Male*. Body length 3.55 mm (n = 1).

Radicle color: same as scape. A5 tyloid: carina-like, not expanded. A11: longer than broad. Upper frons: not hood-like. Frontal depression sculpture: without transverse or oblique carinae below submedian carina. Median longitudinal elevation in frontal depression: present. Major sculpture of gena anteroventrally: rugose; umbilicate-punctate. Major sculpture of gena posteroventrally: rugose; umbilicate-punctate. Microsculpture of gena anteroventrally: absent. Microsculpture of gena posteroventrally: absent. Hyperoccipital carina: wrinkle-like. Median carina extending posteriorly from hyperoccipital carina: absent. Lateral connection between hyperoccipital and occipital carinae: absent. Area between vertex and occipital carina: umbilicate-foveate; rugose. Occipital carina medially: uniformly rounded. Lateral corners of occipital carina: absent.

Mesoscutum anteriorly: not steep. Mesoscutal median carina: absent or incomplete. Major sculpture of mesoscutal midlobe anteriorly: umbilicate-foveate. Major sculpture of mesoscutal midlobe posteriorly: umbilicate-foveate. Microsculpture of mesoscutal midlobe anteriorly: granulate. Microsculpture of mesoscutal midlobe posteriorly: absent. Major sculpture of mesoscutellum: umbilicate-foveate; longitudinally rugose. Microsculpture of mesoscutellum medially: absent. Microsculpture of mesoscutellum laterally: absent. Number of carinae crossing femoral depression: 4 or more. Mesepimeral sulcus pits: more than 5. Setae along anterior limit of femoral depression: arising from tiny pits. Metascutellum dorsally: concave. Metascutellar sculpture centrally: smooth. Metascutellar apex: convex or straight. Metapleuron above ventral metapleural area: crossed by carinae. Lateral propodeal carinae antero-medially: weakly diverging. Metasomal depression setae: present. Anterior areoles of metasomal depression: one or more areoles present. Anterior longitudinal carinae in metasomal depression: absent. Postmarginal vein: present. Hind wing vein (Sc+R): not interrupted.

Carinae between T1 midlobe and T1 lateral carina: absent. T1 midlobe: with 5 longitudinal carinae. Metasomal apex: with no distinct corners.

##### Diagnosis.

Both sexes: Hyperoccipital carina wrinkle-like, not connected to occipital carina laterally or medially. Frontal depression with median carina. Mesoscutellum without granulate sculpture. Metasomal depression setose; lateral propodeal carinae broadly separated. Hind wing Sc+R not interrupted. Male: T7 without acuminate lateral corners.

##### Etymology.

Latin noun, genitive case, meaning “an extension.”

##### Link to distribution map.

[http://hol.osu.edu/map-full.html?id=309296]

##### Material examined.

Holotype, male: **TANZANIA**: Iringa Reg., Kilolo Dist., Udzungwa (Uzungwa) Mts., Luwala (Luwato) Camp area, semi-evergreen montane tropical virgin forest edge, Ndundulu Forest, 1880m, 18.I–25.I.2007, malaise trap, L. A. Hansen & A. Hedayat, OSUC 369369 (deposited in BMNH).

##### Comments.

*Oxyscelio intensionis* bears some resemblance to several Australian species of the *aciculae* group, especially to *Oxyscelio divisionis* Burks. None of the species within that group has a setose metasomal depression.

#### Oxyscelio io

Taxon classificationAnimaliaHymenopteraPlatygastridae

Burks sp. n.

http://zoobank.org/DADCE45A-7345-4893-BB8C-2AF7F2129B31

http://bioguid.osu.edu/xbiod_concepts/309297

Figures 34–39 ; Morphbank ^20^

##### Description.

*Female*. Body length 4.6–5.25 mm (n = 9).

Radicle color: same as scape. A4: broader than long; as long as broad. A5: broader than long. Upper frons: not hood-like. Frontal depression sculpture: with 2–4 complete transverse carinae. Median longitudinal elevation in frontal depression: present. Major sculpture of gena anteroventrally: umbilicate-foveate. Major sculpture of gena posteroventrally: umbilicate-foveate. Microsculpture of gena anteroventrally: absent. Microsculpture of gena posteroventrally: granulate. Hyperoccipital carina: wrinkle-like. Median carina extending posteriorly from hyperoccipital carina: absent; present, anteriorly incomplete. Lateral connection between hyperoccipital and occipital carinae: absent. Area between vertex and occipital carina: umbilicate-foveate; umbilicate-punctate. Occipital carina medially: with nearly flat angular median portion. Lateral corners of occipital carina: sharp and protruding corners present.

Mesoscutum anteriorly: not steep. Mesoscutal median carina: absent or incomplete. Major sculpture of mesoscutal midlobe anteriorly: umbilicate-foveate. Major sculpture of mesoscutal midlobe posteriorly: umbilicate-foveate. Microsculpture of mesoscutal midlobe anteriorly: granulate. Microsculpture of mesoscutal midlobe posteriorly: granulate. Major sculpture of mesoscutellum: umbilicate-foveate. Microsculpture of mesoscutellum medially: granulate. Microsculpture of mesoscutellum laterally: granulate. Number of carinae crossing femoral depression: 4 or more. Mesepimeral sulcus pits: 3–5; more than 5. Setae along anterior limit of femoral depression: arising from tiny pits. Metascutellum dorsally: concave. Metascutellar sculpture centrally: smooth. Metascutellar apex: convex or straight. Metapleuron above ventral metapleural area: crossed by carinae. Lateral propodeal carinae antero-medially: weakly diverging. Metasomal depression setae: absent. Anterior areoles of metasomal depression: one or more areoles present. Anterior longitudinal carinae in metasomal depression: absent. Postmarginal vein: absent. Fore wing apex: reaching apex of T5; reaching middle of T6. Hind wing vein (Sc+R): not interrupted.

Carinae between T1 midlobe and T1 lateral carina: present. T1 midlobe: with 4 longitudinal carinae. T1: without anterior bulge. T6: broader than long; as long as broad. Metasomal apex: rounded. Major sculpture of T6: umbilicate-punctate; longitudinally striate or rugose. Microsculpture of T6: granulate.

*Male*. Body length 4.8 mm (n = 2). A5 tyloid: expanded, teardrop-shaped or sinuate. A11: longer than road. T1 midlobe: with 4 longitudinal carinae. Metasomal apex: with acuminate lateral corners.

##### Diagnosis.

Both sexes: Hyperoccipital carina wrinkle-like, connected to occipital carina by lateral elevation; median carina between hyperoccipital and occipital carinae present but sometimes indicated only posteriorly; occipital carina nearly flat medially. Mesoscutellum with granulate sculpture. Metasomal depression not setose, without median carina; lateral propodeal carinae weakly diverging. Hind wing Sc+R vein complete. Female: T6 rounded apically.

##### Etymology.

Noun, referring to a moon of Jupiter.

##### Link to distribution map.

[http://hol.osu.edu/map-full.html?id=309297]

##### Material examined.

Holotype, female: **GUINEA**: Lola Pref., rainforest, Mount Nimba, 07°41–42'N 08°23'W, 514–740m, XII–1990 – III–1991, flight intercept trap, L. Leblanc, OSUC 369403 (deposited in CNCI). *Paratypes*: (8 females, 3 males) **CAMEROON**: 2 females, OSUC 369362 (BMNH), 369363 (CNCI). **CENTRAL AFRICAN REPUBLIC**: 3 females, 1 male, OSUC 267414, 369392 (OSUC); OSUC 242798, 320839 (SAMC). **CONGO**: 1 female, 1 male, OSUC 470506–470507 (OSUC). **GUINEA**: 1 female, OSUC 369407 (CNCI). **NIGERIA**: 1 male, OSUC 369382 (BMNH). **UGANDA**: 1 female, OSUC 369390 (CNCI).

#### Oxyscelio kylix

Taxon classificationAnimaliaHymenopteraPlatygastridae

Burks sp. n.

http://zoobank.org/420B994F-F83B-421B-9C56-7CDB1A7D2E94

http://bioguid.osu.edu/xbiod_concepts/309298

Figures 40–45 ; Morphbank ^21^

##### Description.

*Female*. Body length 3.3–3.85 mm (n = 13).

Radicle color: same as scape. A4: longer than broad. A5: longer than broad; as long as broad. Upper frons: not hood-like. Frontal depression sculpture: with 1–2 broadly interrupted transverse carinae. Median longitudinal elevation in frontal depression: absent. Major sculpture of gena anteroventrally: umbilicate-foveate. Major sculpture of gena posteroventrally: rugose; umbilicate-punctate. Microsculpture of gena anteroventrally: absent. Microsculpture of gena posteroventrally: granulate. Hyperoccipital carina: complete as a sharp carina. Median carina extending posteriorly from hyperoccipital carina: present, complete; present, anteriorly incomplete. Lateral connection between hyperoccipital and occipital carinae: present as a distinct carina. Area between vertex and occipital carina: rugose; umbilicate-punctate. Occipital carina medially: uniformly rounded. Lateral corners of occipital carina: sharp and protruding corners present.

Mesoscutum anteriorly: not steep. Mesoscutal median carina: present and complete. Major sculpture of mesoscutal midlobe anteriorly: umbilicate-foveate. Major sculpture of mesoscutal midlobe posteriorly: umbilicate-foveate. Microsculpture of mesoscutal midlobe anteriorly: granulate. Microsculpture of mesoscutal midlobe posteriorly: absent; granulate. Major sculpture of mesoscutellum: umbilicate-foveate; obliquely rugose. Microsculpture of mesoscutellum medially: absent. Microsculpture of mesoscutellum laterally: absent. Number of carinae crossing femoral depression: 4 or more. Mesepimeral sulcus pits: more than 5. Setae along anterior limit of femoral depression: arising from rows of foveae. Metascutellum dorsally: concave. Metascutellar sculpture centrally: smooth. Metascutellar apex: convex or straight; shallowly emarginate. Metapleuron above ventral metapleural area: crossed by carinae. Lateral propodeal carinae antero-medially: strongly diverging. Metasomal depression setae: absent. Anterior areoles of metasomal depression: absent. Anterior longitudinal carinae in metasomal depression: absent. Postmarginal vein: present. Fore wing apex: reaching middle of T5. Hind wing vein (Sc+R): not interrupted.

Carinae between T1 midlobe and T1 lateral carina: present. T1 midlobe: obscured by other raised sculpture. T1: with weak anterior bulge that does not closely approach metascutellum. T6: longer than broad. Metasomal apex: rounded. Major sculpture of T6: umbilicate-punctate; longitudinally striate or rugose. Microsculpture of T6: granulate.

*Male*. Body length 3.25–3.65 mm (n = 3). A5 tyloid: carina-like, not expanded. A11: longer than broad. T1 midlobe: with 4 longitudinal carinae. Metasomal apex: with acuminate lateral corners.

##### Diagnosis.

Both sexes: Hyperoccipital carina present and sharp, connected to occipital carina by lateral carina; median carina between hyperoccipital and occipital carinae present but sometimes indicated only posteriorly. Gena with granulate sculpture posteroventrally but not anteroventrally. Mesoscutellum without granulate sculpture. Metasomal depression without setae, without median carina; lateral propodeal carinae strongly diverging. Hind wing Sc+R vein complete. Female: A4 longer than broad.

##### Etymology.

Greek noun, meaning “cup.”

##### Link to distribution map.

[http://hol.osu.edu/map-full.html?id=309298]

##### Material examined.

Holotype, female: **GUINEA**: Lola Pref., Gouan River, rainforest, Mount Nimba, 07°42'N 08°23'W, 514m, 7.I–15.I.1991, flight intercept trap, L. Leblanc, OSUC 369399 (deposited in CNCI). *Paratypes*: (12 females, 3 males) **CAMEROON**: 1 female, OSUC 369364 (BMNH). **CENTRAL AFRICAN REPUBLIC**: 2 females, OSUC 223601, 251693 (SAMC). **CONGO**: 1 male, OSUC 470505 (OSUC). **GABON**: 1 female, OSUC 369395 (BMNH). **GHANA**: 1 female, OSUC 321001 (OSUC). **GUINEA**: 2 females, OSUC 369405, 369411 (CNCI). **IVORY COAST**: 1 female, OSUC 369377 (BMNH). **NIGERIA**: 4 females, 1 male, OSUC 369380–369381 (CNCI); 369378, 369383–369384 (BMNH). **UGANDA**: 1 male, OSUC 369389 (CNCI).

#### Oxyscelio lunae

Taxon classificationAnimaliaHymenopteraPlatygastridae

Burks sp. n.

http://zoobank.org/BD17331A-6B17-4530-B540-05A18128AA85

http://bioguid.osu.edu/xbiod_concepts/309299

Figures 46–51 ; Morphbank ^22^

##### Description.

*Female*. Body length 3.5–3.7 mm (n = 8).

Radicle color: same as scape. A4: longer than broad. A5: broader than long. Upper frons: not hood-like. Frontal depression sculpture: with 3 or more broadly interrupted transverse carinae. Median longitudinal elevation in frontal depression: absent. Major sculpture of gena anteroventrally: umbilicate-foveate. Major sculpture of gena posteroventrally: rugose; umbilicate-punctate. Microsculpture of gena anteroventrally: absent; granulate. Microsculpture of gena posteroventrally: granulate. Hyperoccipital carina: complete as a sharp carina. Median carina extending posteriorly from hyperoccipital carina: present, complete. Lateral connection between hyperoccipital and occipital carinae: present as a distinct carina. Area between vertex and occipital carina: umbilicate-foveate; rugose; umbilicate-punctate. Occipital carina medially: sinuate with a more strongly arched median portion. Lateral corners of occipital carina: sharp and protruding corners present.

Mesoscutum anteriorly: not steep. Mesoscutal median carina: present and complete. Major sculpture of mesoscutal midlobe anteriorly: umbilicate-foveate. Major sculpture of mesoscutal midlobe posteriorly: umbilicate-foveate. Microsculpture of mesoscutal midlobe anteriorly: granulate. Microsculpture of mesoscutal midlobe posteriorly: absent; granulate. Major sculpture of mesoscutellum: umbilicate-foveate. Microsculpture of mesoscutellum medially: absent. Microsculpture of mesoscutellum laterally: granulate. Number of carinae crossing femoral depression: 4 or more. Mesepimeral sulcus pits: more than 5. Setae along anterior limit of femoral depression: arising from rows of foveae. Metascutellum dorsally: concave. Metascutellar sculpture centrally: smooth. Metascutellar apex: convex or straight. Metapleuron above ventral metapleural area: crossed by carinae. Lateral propodeal carinae antero-medially: weakly diverging. Metasomal depression setae: absent. Anterior areoles of metasomal depression: one or more areoles present. Anterior longitudinal carinae in metasomal depression: absent. Postmarginal vein: present. Fore wing apex: reaching middle of T6. Hind wing vein (Sc+R): not interrupted.

Carinae between T1 midlobe and T1 lateral carina: absent. T1 midlobe: with 6 or more longitudinal carinae. T1: without anterior bulge. T6: broader than long; as long as broad. Metasomal apex: rounded; tapering to a sharp point. Major sculpture of T6: umbilicate-punctate. Microsculpture of T6: absent.

*Male*. Body length 3.4–3.65 mm (n = 20). A5 tyloid: carina-like, not expanded. A11: longer than broad. T1 midlobe: with 4 longitudinal carinae. Metasomal apex: with acuminate lateral corners.

##### Diagnosis.

Both sexes: Hyperoccipital carina present and sharp, connected to occipital carina by lateral carina; median carina present between hyperoccipital and occipital carinae. Gena with granulate sculpture posteroventrally but not anteroventrally. Mesoscutellum with granulate sculpture laterally. Metasomal depression not setose, without median carina; lateral propodeal carinae weakly diverging. Hind wing Sc+R vein complete. Female: A4 longer than broad.

##### Etymology.

Latin noun, genitive case, meaning “moon.”

##### Link to distribution map.

[http://hol.osu.edu/map-full.html?id=309299]

##### Material examined.

Holotype, female: **CAMEROON**: Nkoemvom, VIII–1980, malaise trap, D. Jackson, OSUC 369365 (deposited in BMNH). *Paratypes*: (23 females, 57 males) **CAMEROON**: 15 males, OSUC 369340, 369342–369346, 369360 (CNCI), OSUC 369341, 369347–369352, 369359 (BMNH). **CENTRAL AFRICAN REPUBLIC**: 20 females, 27 males, OSUC 369391, 223802, 242799, 282894, 282896, 320854, 369393 (CNCI); OSUC 176083, 218855, 233095–233096, 320840–320841, 320845, 320847, 320849–320853, 320855, 369385, 369394 (OSUC); OSUC 176091, 218850, 223639, 223801, 225982–225985, 251694–251698, 267415–267417, 282879, 282895, 317893, 320838, 320842–320844, 320846, 320848 (SAMC). **DEMOCRATIC REPUBLIC OF THE CONGO**: 6 males, OSUC 369335–369339 (CNCI); OSUC 268178 (USNM). **GHANA**: 4 males, OSUC 369386–369387 (CNCI); OSUC 435286 (OSUC). **GUINEA**: 1 female, 5 males, OSUC 369400–369401, 369404, 369406, 369408–369409 (CNCI). **SIERRA LEONE**: 1 female, OSUC 462603 (MZLU). **TOGO**: 1 female, OSUC 320828 (BMNH).

##### Comments.

*Oxyscelio lunae* is by far the most commonly collected species of its genus from Africa, although nearly all known specimens are male. It is very similar to *Oxyscelio pulveris*, but after extensive comparison of the two series we concluded that they are different species. The chief difference is the considerably more granulate sculpture of *Oxyscelio pulveris*, which occurs in conjunction with lower and more rounded (therefore, less sharp and less distinctive) carinae, especially the hyperoccipital and mesoscutellar disc carinae. These features are accompanied by some more vague and less easily described differences in eye shape, mesosomal and metasomal sclerite shape, and metasomal sculpture.

#### Oxyscelio nemesis

Taxon classificationAnimaliaHymenopteraPlatygastridae

Burks sp. n.

http://zoobank.org/378ACB70-2B66-476E-8E7C-1F4AA4856FFC

http://bioguid.osu.edu/xbiod_concepts/312620

Figures 52–57 ; Morphbank ^23^

##### Description.

Female. Body length 4.8 mm (n = 1).

Radicle color: darker than scape. A4: broader than long. A5: broader than long. Upper frons: not hood-like. Frontal depression sculpture: without transverse or oblique carinae below submedian carina. Median longitudinal elevation in frontal depression: absent. Major sculpture of gena anteroventrally: umbilicate-foveate. Major sculpture of gena posteroventrally: rugose; umbilicate-punctate. Microsculpture of gena anteroventrally: absent. Microsculpture of gena posteroventrally: granulate. Hyperoccipital carina: wrinkle-like. Median carina extending posteriorly from hyperoccipital carina: absent. Lateral connection between hyperoccipital and occipital carinae: absent. Area between vertex and occipital carina: umbilicate-foveate; umbilicate-punctate. Occipital carina medially: with nearly flat angular median portion. Lateral corners of occipital carina: sharp and protruding corners present.

Mesoscutum anteriorly: steep. Mesoscutal median carina: absent or incomplete. Major sculpture of mesoscutal midlobe anteriorly: umbilicate-foveate. Major sculpture of mesoscutal midlobe posteriorly: umbilicate-foveate. Microsculpture of mesoscutal midlobe anteriorly: granulate. Microsculpture of mesoscutal midlobe posteriorly: granulate. Major sculpture of mesoscutellum: umbilicate-foveate. Microsculpture of mesoscutellum medially: granulate. Microsculpture of mesoscutellum laterally: granulate. Number of carinae crossing femoral depression: 4 or more. Mesepimeral sulcus pits: more than 5. Setae along anterior limit of femoral depression: arising from rows of foveae. Metascutellum dorsally: flat or convex. Metascutellar sculpture centrally: rugose. Metascutellar apex: convex or straight. Metapleuron above ventral metapleural area: crossed by carinae; foveate or rugose. Lateral propodeal carinae antero-medially: weakly diverging. Metasomal depression setae: present. Anterior areoles of metasomal depression: absent. Anterior longitudinal carinae in metasomal depression: absent. Postmarginal vein: absent. Fore wing apex: reaching apex of T5. Hind wing vein (Sc+R): not interrupted.

Carinae between T1 midlobe and T1 lateral carina: absent. T1 midlobe: with 4 longitudinal carinae. T1: without anterior bulge. T6: longer than broad. Metasomal apex: rounded. Major sculpture of T6: umbilicate-punctate. Microsculpture of T6: granulate.

##### Diagnosis.

Both sexes: Hyperoccipital carina wrinkle-like, not connected to occipital carina laterally; median carina between hyperoccipital and occipital carinae absent; occipital carina nearly flat medially. Mesoscutellum with granulate sculpture. Metasomal depression setose, without median carina; lateral propodeal carinae weakly diverging. Hind wing Sc+R vein complete. Female: T6 rounded apically.

##### Etymology.

Latin noun, genitive case.

##### Link to distribution map.

[http://hol.osu.edu/map-full.html?id=312620]

##### Material examined.

Holotype, female: **NIGERIA**: Oyo St., International Institute of Tropical Agriculture (IITA), Ibadan, XI–1987, pan trap, J. S. Noyes, OSUC 369379 (deposited in BMNH).

##### Comments.

*Oxyscelio nemesis* strongly resembles *Oxyscelio io*, and they both vaguely resemble *Oxyscelio teli*. These three species together may form a monophyletic species complex, but such a grouping would currently be difficult to fully distinguish from similar African species. It can be roughly defined by the medially more or less flat occipital carina, but this feature in *Oxyscelio io* is variable and sometimes not greatly different from that of some excluded African species.

The shape of the head of this species and the carinate margin of the antennal scribe make it superficially similar to the genus *Baryconus* Förster. The fore wing venation, however, makes it clear that this is an *Oxyscelio*: it lacks elongate marginal and postmarginal veins, and the stigma vein arises from the upturned apical portion of the submarginal vein.

#### Oxyscelio pulveris

Taxon classificationAnimaliaHymenopteraPlatygastridae

Burks sp. n.

http://zoobank.org/307734E3-F87F-44F8-A004-E828FB52908B

http://bioguid.osu.edu/xbiod_concepts/309300

Figures 58–61 ; Morphbank ^24^

##### Description.

*Female*. Body length 3.5 mm (n = 1).

Radicle color: same as scape. A4: longer than broad. A5: broader than long. Upper frons: not hood-like. Frontal depression sculpture: with 3 or more broadly interrupted transverse carinae. Median longitudinal elevation in frontal depression: absent. Major sculpture of gena anteroventrally: umbilicate-foveate. Major sculpture of gena posteroventrally: umbilicate-punctate. Microsculpture of gena anteroventrally: granulate. Microsculpture of gena posteroventrally: granulate. Hyperoccipital carina: complete as a sharp carina. Median carina extending posteriorly from hyperoccipital carina: present, complete. Lateral connection between hyperoccipital and occipital carinae: present as a distinct carina. Area between vertex and occipital carina: rugose; umbilicate-punctate. Occipital carina medially: sinuate with a more strongly arched median portion. Lateral corners of occipital carina: sharp and protruding corners present.

Mesoscutum anteriorly: not steep. Mesoscutal median carina: present and complete. Major sculpture of mesoscutal midlobe anteriorly: umbilicate-foveate. Major sculpture of mesoscutal midlobe posteriorly: umbilicate-foveate. Microsculpture of mesoscutal midlobe anteriorly: granulate. Microsculpture of mesoscutal midlobe posteriorly: absent; granulate. Major sculpture of mesoscutellum: umbilicate-foveate. Microsculpture of mesoscutellum medially: granulate. Microsculpture of mesoscutellum laterally: granulate. Number of carinae crossing femoral depression: 4 or more. Mesepimeral sulcus pits: more than 5. Setae along anterior limit of femoral depression: arising from rows of foveae. Metascutellum dorsally: concave. Metascutellar sculpture centrally: smooth. Metascutellar apex: convex or straight. Metapleuron above ventral metapleural area: crossed by carinae. Lateral propodeal carinae antero-medially: weakly diverging. Metasomal depression setae: absent. Anterior areoles of metasomal depression: one or more areoles present. Anterior longitudinal carinae in metasomal depression: median carina present. Postmarginal vein: present. Fore wing apex: reaching beyond T6. Hind wing vein (Sc+R): not interrupted.

Carinae between T1 midlobe and T1 lateral carina: absent. T1 midlobe: with 5 longitudinal carinae. T1: without anterior bulge. T6: longer than broad. Metasomal apex: tapering to a sharp point. Major sculpture of T6: longitudinally striate or rugose. Microsculpture of T6: granulate.

##### Diagnosis.

Both sexes: Hyperoccipital carina present and sharp, connected to occipital carina by lateral carina; median carina present between hyperoccipital and occipital carinae. Gena with granulate sculpture posteroventrally but not anteroventrally. Mesoscutellum with granulate sculpture throughout. Metasomal depression without setae, with median carina; lateral propodeal carinae weakly diverging. Hind wing Sc+R vein complete. Female: A4 longer than broad.

##### Etymology.

Latin noun, genitive case, meaning “dust.”

##### Link to distribution map.

[http://hol.osu.edu/map-full.html?id=309300]

##### Material examined.

Holotype, female: **RWANDA**: primary rainforest, Nyungwe Forest, 02°46’10”S 29°21’09”E, 24.VIII–26.VIII.1993, pan trap/flight intercept trap/malaise trap, L. Leblanc, OSUC 369388 (deposited in CNCI).

##### Comments.

See the discussion under *Oxyscelio lunae* for comparison of these two very similar species.

#### Oxyscelio quassus

Taxon classificationAnimaliaHymenopteraPlatygastridae

Burks sp. n.

http://zoobank.org/372D126A-218D-4044-B4AD-F5C84DEC6924

http://bioguid.osu.edu/xbiod_concepts/309301

Figures 62–67 ; Morphbank ^25^

##### Description.

*Female*. Body length 2.95–3.55 mm (n = 4).

Radicle color: same as scape. A4: longer than broad. A5: longer than broad. Upper frons: not hood-like. Frontal depression sculpture: with 1–2 broadly interrupted transverse carinae. Median longitudinal elevation in frontal depression: absent. Major sculpture of gena anteroventrally: umbilicate-foveate. Major sculpture of gena posteroventrally: absent; umbilicate-punctate. Microsculpture of gena anteroventrally: granulate. Microsculpture of gena posteroventrally: granulate. Hyperoccipital carina: wrinkle-like. Median carina extending posteriorly from hyperoccipital carina: absent. Lateral connection between hyperoccipital and occipital carinae: absent. Area between vertex and occipital carina: umbilicate-foveate; rugose. Occipital carina medially: uniformly rounded. Lateral corners of occipital carina: absent.

Mesoscutum anteriorly: not steep. Mesoscutal median carina: absent or incomplete. Major sculpture of mesoscutal midlobe anteriorly: umbilicate-foveate. Major sculpture of mesoscutal midlobe posteriorly: umbilicate-foveate. Microsculpture of mesoscutal midlobe anteriorly: granulate. Microsculpture of mesoscutal midlobe posteriorly: granulate. Major sculpture of mesoscutellum: umbilicate-foveate; longitudinally rugose. Microsculpture of mesoscutellum medially: absent. Microsculpture of mesoscutellum laterally: absent. Number of carinae crossing femoral depression: 4 or more. Mesepimeral sulcus pits: more than 5. Setae along anterior limit of femoral depression: arising from rows of foveae. Metascutellum dorsally: concave. Metascutellar sculpture centrally: smooth. Metascutellar apex: shallowly emarginate. Metapleuron above ventral metapleural area: foveate or rugose. Lateral propodeal carinae antero-medially: weakly diverging. Metasomal depression setae: present. Anterior areoles of metasomal depression: one or more areoles present. Anterior longitudinal carinae in metasomal depression: absent. Postmarginal vein: present. Fore wing apex: reaching middle of T6; reaching apex of T6. Hind wing vein (Sc+R): not interrupted.

Carinae between T1 midlobe and T1 lateral carina: absent. T1 midlobe: with 5 longitudinal carinae. T1: without anterior bulge. T6: broader than long. Metasomal apex: rounded. Major sculpture of T6: umbilicate-punctate. Microsculpture of T6: granulate.

*Male*. Body length 2.7–3.15 mm (n = 5). A5 tyloid: carina-like, not expanded. A11: longer than broad. T1 midlobe: with 4 longitudinal carinae. Metasomal apex: with acuminate lateral corners.

##### Diagnosis.

Both sexes: Hyperoccipital carina wrinkle-like, not connected to occipital carina laterally; median carina between hyperoccipital and occipital carinae absent. Gena with granulate sculpture anteroventrally and posteroventrally. Mesoscutellum without granulate sculpture. Metasomal depression setose, without median carina; lateral propodeal carinae weakly diverging. Hind wing Sc+R vein complete. Female: A4 longer than broad.

##### Etymology.

Latin noun (4th declension), genitive case, meaning “the act of shaking.”

##### Link to distribution map.

[http://hol.osu.edu/map-full.html?id=309301]

##### Material examined.

Holotype, female: **GUINEA**: Lola Pref., Gouan River, rainforest, Mount Nimba, 07°42'N 08°23'W, 514m, 7.I–15.I.1991, flight intercept trap, L. Leblanc, OSUC 369398 (deposited in CNCI). *Paratypes*: (3 females, 5 males) **CAMEROON**: 1 female, OSUC 369361 (BMNH). **GHANA**: 1 male, OSUC 429536 (OSUC). **GUINEA**: 4 males, OSUC 369396–369397, 369402, 369410 (CNCI). **SOMALIA**: 2 females, OSUC 369412–369413 (CNCI).

#### Oxyscelio teli

Taxon classificationAnimaliaHymenopteraPlatygastridae

Burks sp. n.

http://zoobank.org/936A2811-698F-49A3-8ACF-C3DCA5D61F7F

http://bioguid.osu.edu/xbiod_concepts/309304

Figures 68–71 ; Morphbank ^26^

##### Description.

*Female*. Body length 3.2–3.35 mm (n = 4).

Radicle color: same as scape; darker than scape. A4: broader than long; as long as broad. A5: broader than long. Upper frons: not hood-like. Frontal depression sculpture: with 2–4 complete transverse carinae. Median longitudinal elevation in frontal depression: absent. Major sculpture of gena anteroventrally: umbilicate-foveate; rugose. Major sculpture of gena posteroventrally: umbilicate-foveate; rugose. Microsculpture of gena anteroventrally: absent; granulate. Microsculpture of gena posteroventrally: granulate. Hyperoccipital carina: wrinkle-like. Median carina extending posteriorly from hyperoccipital carina: present, complete. Lateral connection between hyperoccipital and occipital carinae: present as a rounded elevation. Area between vertex and occipital carina: rugose; umbilicate-punctate. Occipital carina medially: with nearly flat angular median portion. Lateral corners of occipital carina: sharp and protruding corners present.

Mesoscutum anteriorly: steep. Mesoscutal median carina: present and complete. Major sculpture of mesoscutal midlobe anteriorly: umbilicate-foveate. Major sculpture of mesoscutal midlobe posteriorly: umbilicate-foveate. Microsculpture of mesoscutal midlobe anteriorly: granulate. Microsculpture of mesoscutal midlobe posteriorly: absent. Major sculpture of mesoscutellum: umbilicate-foveate. Microsculpture of mesoscutellum medially: absent. Microsculpture of mesoscutellum laterally: absent. Number of carinae crossing femoral depression: 4 or more. Mesepimeral sulcus pits: more than 5. Setae along anterior limit of femoral depression: arising from rows of foveae. Metascutellum dorsally: concave. Metascutellar sculpture centrally: smooth. Metascutellar apex: shallowly emarginate. Metapleuron above ventral metapleural area: foveate or rugose. Lateral propodeal carinae antero-medially: weakly diverging. Metasomal depression setae: absent. Anterior areoles of metasomal depression: absent. Anterior longitudinal carinae in metasomal depression: absent. Postmarginal vein: present. Fore wing apex: reaching middle of T5; reaching apex of T5. Hind wing vein (Sc+R): not interrupted.

Carinae between T1 midlobe and T1 lateral carina: absent. T1 midlobe: with 5 longitudinal carinae. T1: without anterior bulge. T6: broader than long. Metasomal apex: tapering to a sharp point. Major sculpture of T6: umbilicate-punctate. Microsculpture of T6: absent; granulate.

##### Diagnosis.

Both sexes: Hyperoccipital carina wrinkle-like, connected to occipital carina by lateral elevation; median carina between hyperoccipital and occipital carinae present. Mesoscutellum without granulate sculpture. Metasomal depression without setae, without median carina; lateral propodeal carinae weakly diverging. Hind wing Sc+R vein complete. Female: A4 broader than long; T6 sharply acuminate apically.

##### Etymology.

Latin noun, genitive case, meaning “dart.”

##### Link to distribution map.

[http://hol.osu.edu/map-full.html?id=309304]

##### Material examined.

Holotype, female: **KENYA**: Eastern Prov., nr. Ewaso Ngiro River, next to headquarters, riverine forest, Samburu National Reserve, 00.56797°N 37.53563°E, 874m, 18.IX–2.X.2007, malaise trap, R. Copeland, OSUC 381658 (deposited in NMKE). *Paratypes*: **KENYA**: 3 females, OSUC 381659 (NMKE); OSUC 381657 (OSUC); OSUC 381657 (USNM).

#### Oxyscelio xenii

Taxon classificationAnimaliaHymenopteraPlatygastridae

Burks sp. n.

http://zoobank.org/AB2F63F2-0E61-4C82-9469-3D242ED29D44

http://bioguid.osu.edu/xbiod_concepts/309302

Figures 72–77 ; Morphbank ^27^

##### Description.

*Female*. Body length 3.15–3.35 mm (n = 2).

Radicle color: darker than scape. A4: longer than broad. A5: broader than long. Upper frons: not hood-like. Frontal depression sculpture: with 2–4 complete transverse carinae. Median longitudinal elevation in frontal depression: absent. Major sculpture of gena anteroventrally: umbilicate-foveate; rugose. Major sculpture of gena posteroventrally: absent; rugose. Microsculpture of gena anteroventrally: granulate. Microsculpture of gena posteroventrally: granulate. Hyperoccipital carina: wrinkle-like. Median carina extending posteriorly from hyperoccipital carina: absent. Lateral connection between hyperoccipital and occipital carinae: absent. Area between vertex and occipital carina: umbilicate-foveate; rugose. Occipital carina medially: uniformly rounded. Lateral corners of occipital carina: absent.

Mesoscutum anteriorly: not steep. Mesoscutal median carina: absent or incomplete. Major sculpture of mesoscutal midlobe anteriorly: umbilicate-foveate. Major sculpture of mesoscutal midlobe posteriorly: umbilicate-foveate. Microsculpture of mesoscutal midlobe anteriorly: granulate. Microsculpture of mesoscutal midlobe posteriorly: absent; granulate. Major sculpture of mesoscutellum: umbilicate-foveate. Microsculpture of mesoscutellum medially: punctate. Microsculpture of mesoscutellum laterally: punctate. Number of carinae crossing femoral depression: 4 or more. Mesepimeral sulcus pits: more than 5. Setae along anterior limit of femoral depression: arising from rows of foveae. Metascutellum dorsally: concave. Metascutellar sculpture centrally: smooth. Metascutellar apex: convex or straight. Metapleuron above ventral metapleural area: foveate or rugose. Lateral propodeal carinae antero-medially: weakly diverging. Metasomal depression setae: present. Anterior areoles of metasomal depression: one or more areoles present. Anterior longitudinal carinae in metasomal depression: absent. Postmarginal vein: present. Fore wing apex: reaching middle of T6; reaching apex of T6. Hind wing vein (Sc+R): interrupted.

Carinae between T1 midlobe and T1 lateral carina: absent. T1 midlobe: with 6 or more longitudinal carinae. T1: without anterior bulge. T6: broader than long. Metasomal apex: rounded. Major sculpture of T6: umbilicate-punctate. Microsculpture of T6: granulate.

##### Diagnosis.

Both sexes: Hyperoccipital carina wrinkle-like, not connected to occipital carina laterally or medially. Gena with granulate sculpture anteroventrally and posteroventrally. Mesoscutellum without granulate sculpture, with some punctate sculpture between foveae. Metasomal depression setose, without median carina; lateral propodeal carinae weakly diverging. Hind wing Sc+R interrupted. T1 without carinae between midlobe and lateral carina. Female: A4 longer than broad.

##### Etymology.

Latin noun, genitive case, meaning “a present intended for a guest.”

##### Link to distribution map.

[http://hol.osu.edu/map-full.html?id=309302]

##### Material examined.

Holotype, female: **MALAWI**: Chitipa Dist., 18km SSE Chisenga, Jembya Forest Reserve, 10°08'S 33°27'E, 1870m, 1.I–10.I.1989, J. Rawlins & S. Thompson, OSUC 369376 (deposited in CNCI). *Paratype*: **MALAWI**: 1 female, OSUC 369375 (CNCI).

## Supplementary Material

XML Treatment for Oxyscelio

XML Treatment for Oxyscelio absentiae

XML Treatment for Oxyscelio bicolor

XML Treatment for Oxyscelio galeri

XML Treatment for Oxyscelio gyri

XML Treatment for Oxyscelio idoli

XML Treatment for Oxyscelio intensionis

XML Treatment for Oxyscelio io

XML Treatment for Oxyscelio kylix

XML Treatment for Oxyscelio lunae

XML Treatment for Oxyscelio nemesis

XML Treatment for Oxyscelio pulveris

XML Treatment for Oxyscelio quassus

XML Treatment for Oxyscelio teli

XML Treatment for Oxyscelio xenii

## Endnotes

2
http://biocol.org/urn:lsid:biocol.org:col:1009

3
http://biocol.org/urn:lsid:biocol.org:col:1012

4
http://biocol.org/urn:lsid:biocol.org:col:33453

5
http://biocol.org/urn:lsid:biocol.org:col:33791

6
http://biocol.org/urn:lsid:biocol.org:col:33943

7
http://grbio.org/cool/h6fg-emd8

8
http://biocol.org/urn:lsid:biocol.org:col:1014

9
http://biocol.org/urn:lsid:biocol.org:col:34157

10
http://biocol.org/urn:lsid:biocol.org:col:34161

11
http://biocol.org/urn:lsid:biocol.org:col:1018

12
http://biocol.org/urn:lsid:biocol.org:col:1019

13
http://biocol.org/urn:lsid:biocol.org:col:34593

14
http://www.morphbank.net/?id=854135

15
http://www.morphbank.net/?id=854136

16
http://www.morphbank.net/?id=854137

17
http://www.morphbank.net/?id=854138

18
http://www.morphbank.net/?id=854139

19
http://www.morphbank.net/?id=854140

20
http://www.morphbank.net/?id=854141

21
http://www.morphbank.net/?id=854142

22
http://www.morphbank.net/?id=854143

23
http://www.morphbank.net/?id=854144

24
http://www.morphbank.net/?id=854145

25
http://www.morphbank.net/?id=854146

26
http://www.morphbank.net/?id=854147

27
http://www.morphbank.net/?id=854148

## Appendix I

Characters. * = used in phylogenetic analysis.

1. Radicle color

1. same as scape

2. darker than scape

2. A4 (female)

1. broader than long

2. longer than broad

3. as long as broad

3. A5 (female)

1. broader than long

2. longer than broad

3. as long as broad

4. Upper frons

1. not hood-like

2. hood-like, protruding over pedicel when antenna at rest

5. Frontal depression sculpture

1. without transverse or oblique carinae below submedian carina

2. with 3 or more broadly interrupted transverse carinae

3. with 2–4 complete transverse carinae

4. with 1–2 broadly interrupted transverse carinae

6. Median longitudinal elevation in frontal depression

1. absent

2. present

7. Major sculpture of gena anteroventrally

1. umbilicate-foveate

2. rugose

3. umbilicate-punctate

8. Major sculpture of gena posteroventrally

1. absent

2. umbilicate-foveate

3. rugose

4. umbilicate-punctate

9. Microsculpture of gena anteroventrally

1. absent

2. granulate

10. Microsculpture of gena posteroventrally

1. absent

2. granulate

11. Hyperoccipital carina*

1. complete as a sharp carina

2. not indicated medially

3. wrinkle-like

12. Median carina extending posteriorly from hyperoccipital carina*

1. absent

2. present, complete

3. present, anteriorly incomplete

13. Lateral connection between hyperoccipital and occipital carinae*

1. absent

2. present as a distinct carina

3. present as a rounded elevation

14. Area between vertex and occipital carina

1. umbilicate-foveate

2. rugose

3. umbilicate-punctate

15. Occipital carina medially*

1. uniformly rounded

2. sinuate with a more strongly arched median portion

3. with nearly flat angular median portion

16. Lateral corners of occipital carina*

1. absent

2. sharp and protruding corners present

17. Mesoscutum anteriorly

1. steep

2. not steep

18. Mesoscutal median carina

1. present and complete

2. absent or incomplete

19. Major sculpture of mesoscutal midlobe anteriorly

1. umbilicate-foveate

2. umbilicate-punctate

20. Major sculpture of mesoscutal midlobe posteriorly

1. umbilicate-foveate

2. transversely rugose

3. obliquely rugose

4. longitudinally rugose

21. Microsculpture of mesoscutal midlobe anteriorly

1. granulate

22. Microsculpture of mesoscutal midlobe posteriorly

1. absent

2. granulate

23. Major sculpture of mesoscutellum

1. umbilicate-foveate

2. longitudinally rugose

3. obliquely rugose

24. Microsculpture of mesoscutellum medially

1. absent

2. granulate

3. punctate

25. Microsculpture of mesoscutellum laterally

1. absent

2. granulate

3. punctate

26. Number of carinae crossing femoral depression

1. more than 5

27. Mesepimeral sulcus pits

1. 3–5

2. more than 5

28. Setae along anterior limit of femoral depression

1. arising from rows of foveae

2. arising from tiny pits

29. Metascutellum dorsally

1. concave

2. flat or convex

30. Metascutellar sculpture centrally

1. smooth

2. rugose

3. with longitudinal carinae

4. foveate

31. Metascutellar apex

1. convex or straight

2. deeply emarginate

3. sharply acuminate

4. shallowly emarginate

32. Metapleuron above ventral metapleural area

1. crossed by carinae

2. foveate or rugose

33. Lateral propodeal carinae antero-medially (female)

1. strongly diverging

2. weakly diverging

34. Metasomal depression setae*

1. absent

2. present

35. Anterior areoles of metasomal depression

1. absent

2. one or more areoles present

36. Anterior longitudinal carinae in metasomal depression

1. absent

2. median carina present

37. Postmarginal vein*

1. present

2. absent

38. Fore wing apex (female)

1. reaching middle of T5

2. reaching apex of T5

3. reaching middle of T6

4. reaching apex of T6

5. reaching beyond T6

39. Hind wing vein (Sc+R)*

1. not interrupted

2. interrupted

40. Carinae between T1 midlobe and T1 lateral carina*

1. present

2. absent

41. T1 midlobe (female)*

1. with 4 longitudinal carinae

2. with 5 longitudinal carinae

3. with 6 or more longitudinal carinae

4. obscured by other raised sculpture

42. T1 (female)*

1. without anterior bulge

2. with long anterior bulge that reaches metascutellum

3. with weak anterior bulge that does not closely approach metascutellum

43. T6

1. broader than long

2. longer than broad

3. as long as broad

44. Metasomal apex (female)*

1. rounded

2. tapering to a sharp point

45. Major sculpture of T6

1. umbilicate-punctate

2. longitudinally striate or rugose

46. Microsculpture of T6

1. absent

2. granulate

47. A5 tyloid

1. carina-like, not expanded

2. expanded, teardrop-shaped or sinuate

48. A11 (male)*

1. longer than broad

49. T1 midlobe (male)

1. with 4 longitudinal carinae

2. with 5 longitudinal carinae

3. with 6 or more longitudinal carinae

50. Metasomal apex (male)*

1. with acuminate lateral corners

2. with no distinct corners

## Matrix

**Matrix:**

	1	2	3	4	5	6	7	8	9	10	11	12	13	14
*Bracalba cuneata*	1	0	0	0	0	0	0	0	0	2	0	0	0	1
*Oxyscelio absentiae*	1	0	0	0	0	0	0	1	0	3	1	0	?	?
*Oxyscelio bicolor*	2	0	0	0	0	1	0	1	0	2	0	0	0	0
*Oxyscelio galeri*	0	0	0	1	0	0	0	0	1	?	?	?	0	1
*Oxyscelio gyri*	0	0	0	0	0	0	0	0	0	[12]	0	0	0	0
*Oxyscelio idoli*	2	0	0	0	0	0	0	1	0	2	0	0	0	0
*Oxyscelio intensionis*	2	0	0	0	0	1	0	0	1	?	?	?	0	1
*Oxyscelio io*	2	[02]	0	2	1	0	1	0	0	0	0	0	0	0
*Oxyscelio kylix*	0	[12]	1	0	1	0	0	0	0	3	2	0	0	0
*Oxyscelio lunae*	0	1	1	1	1	0	0	0	1	2	0	[01]	0	0
*Oxyscelio nemesis*	2	0	0	2	1	1	1	0	1	0	0	0	?	?
*Oxyscelio pulveris*	0	1	1	1	1	0	0	0	1	1	0	1	?	?
*Oxyscelio quassus*	2	0	0	0	0	1	0	0	1	1	0	0	0	0
*Oxyscelio teli*	2	1	2	2	1	0	0	0	1	1	0	1	?	?
*Oxyscelio xenii*	2	0	0	0	0	1	0	1	1	2	0	0	?	?

## Supplementary materials

Supplementary material 1 Taxonomic records used in the present paper. Data type: specimen data Explanation note: File format: DarwinCore Archive. This dataset is made available under the Open Database License (http://opendatacommons.org/licenses/odbl/1.0/). The Open Database License (ODbL) is a license agreement intended to allow users to freely share, modify, and use this Dataset while maintaining this same freedom for others, provided that the original source and author(s) are credited.Roger A. Burks, Lubomír Masner, Norman F. Johnson, Andrew D. Austin

Supplementary material 2 Locality records used in the present paper. Data type: specimen data Explanation note: File format: DarwinCore Archive. This dataset is made available under the Open Database License (http://opendatacommons.org/licenses/odbl/1.0/). The Open Database License (ODbL) is a license agreement intended to allow users to freely share, modify, and use this Dataset while maintaining this same freedom for others, provided that the original source and author(s) are credited.Roger A. Burks, Lubomír Masner, Norman F. Johnson, Andrew D. Austin
